# Bridging knowledge gaps in two trematode life cycles: insights from southern African freshwater ecosystems

**DOI:** 10.1016/j.ijppaw.2026.101205

**Published:** 2026-02-13

**Authors:** Annabell Hüsken, Marliese Truter, Jessica Schwelm, Bernd Sures, Nico J. Smit, Wynand Malherbe

**Affiliations:** a Aquatic Ecology and Centre for Water and Environmental Research, University of Duisburg-Essen, Universitätsstraße 5, 45141, Essen, Germany; b Research Center One Health Ruhr, Research Alliance Ruhr, University of Duisburg-Essen, Universitätsstraße 5, 45141, Essen, Germany; c Water Research Group, Unit for Environmental Sciences and Management, North-West University – Potchefstroom Campus, Potchefstroom, 2520, South Africa; d South African Institute for Aquatic Biodiversity (NRF-SAIAB), Makhanda, 6140, South Africa

**Keywords:** Aquatic parasites, Life cycle, Echinostomatidae, Diplostomidae, Metacercariae, Cercariae

## Abstract

Understanding and resolving trematode life cycles is increasingly recognised as an important objective in helminth research, as many species are known only from certain developmental stages or hosts. For the diverse but incompletely documented trematode fauna of southern Africa, life cycle data are particularly scarce. This study aimed to reconstruct trematode life cycles by investigating conspecific developmental stages in first intermediate snail and second intermediate fish hosts across South Africa and Zambia. Snails (*Bulinus* sp.) and fishes [*Enteromius oraniensis* (Barnard, 1943) and *Clarias gariepinus* (Burchell, 1822)] were collected between 2019 and 2025. Molecular sequencing (28S rDNA, ITS1–5.8S–ITS2, *nad*1 and *cox*1) confirmed conspecificity among developmental stages of two trematode taxa: previously published *Petasiger* sp. 5 (Echinostomatidae) and *Tylodelphys* sp. 2 (Diplostomidae). Cercariae and metacercariae of both lineages were morphologically examined and descriptions provided. For *Petasiger* sp. 5, life cycle reconstruction linked cercariae from *Bulinus* sp. with metacercariae infecting the gills of *E. oraniensis*, representing the first confirmed record of *Petasiger* sp. 5 metacercariae infecting cyprinids of the Smiliogastrinae in Africa. For *Tylodelphys* sp. 2, we provide the first record of its cercarial stage and identify *Bulinus* sp. as the first intermediate host, genetically linking it to metacercariae recorded from the cranial cavity of *C. gariepinus*. These findings underscore the value of molecular matching for life cycle reconstruction of digenean trematodes. By elucidating previously unknown developmental stages and host associations of *Petasiger* sp. 5 and *Tylodelphys* sp. 2, this study advances the understanding of trematode diversity and transmission in African freshwater ecosystems and provides essential data on first and second intermediate host stages for future species descriptions of these taxa.

## Highlights

• Life cycle reconstruction of two aquatic southern African trematode species.
• Molecular data linked different larval and adult stages.
• First record of *Petasiger* sp. 5 metacercariae in African fishes.

## Graphical abstract

**Figure:**
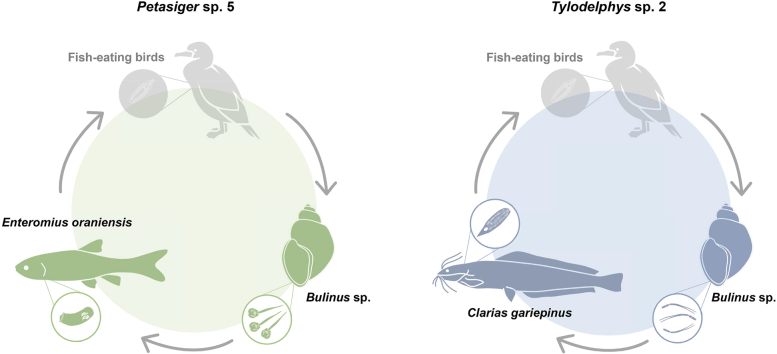

## 1 Introduction

The evolution of complex life cycles in helminth parasites has produced a remarkable diversity of species that sequentially exploit invertebrate and vertebrate hosts through distinct developmental life stages, each morphologically and functionally adapted to their respective host (Sures et al., 2025). Although research on helminth taxonomy and diversity has increased substantially in recent decades, the linkage of developmental stages and, consequently, the elucidation of life cycles lags behind (Blasco-Costa and Poulin, 2017; Poulin, 2025). As a result, many species remain known only from certain life stages or hosts, without integration into the larger context of their life cycles. Yet, comprehensive understanding of parasite life cycles is crucial for fundamentally resolving species taxonomy, ecology and evolution, for instance with regard to host specificity, transmission strategies, predator-prey interactions and food web dynamics (Blasco-Costa and Poulin, 2017).

Digenean trematodes constitute the most diverse group of helminth parasites, with a cosmopolitan distribution and an estimated diversity ranging from 24,000 to more than 180,000 species (Dobson et al., 2008; Carlson et al., 2020). Although approximately 18,000 species have been formally described, complete life cycles are known only for a fraction of them (Bray et al., 2008; Blasco-Costa and Poulin, 2017). Digenean trematodes exemplify the concept of complex life cycles, as they typically exploit three hosts in succession, although variation from one to four hosts is known (Poulin and Cribb, 2002; Sures et al., 2025). Sexual adults (maritae) reproduce in vertebrate definitive hosts, releasing eggs into the environment. Infection of the first intermediate host, typically molluscs, occurs via ingestion of eggs or penetration by hatched miracidial larvae. Within the mollusc, asexual reproduction by a mother sporocyst produces daughter sporocysts or rediae (parthenitae), which in turn produce free-swimming cercariae that actively seek the second intermediate host. After penetration, cercariae encyst as metacercariae in or on this host, commonly molluscs, fishes, crustaceans, amphibians, or aquatic insects, where they persist until trophic transmission to the definitive host completes the life cycle (Sures et al., 2025). Historically, the distinct morphological characteristics associated with each developmental stage often led to separate descriptions and naming of trematode cercariae and metacercariae (Cribb et al., 2025), and many such records remain unlinked to up- or downstream life stages until today (Hechinger, 2023). However, advances in DNA sequencing and increasing availability of reference data have enabled matching of life stages not only through morphology and experimental infections, but also genetic data from suitable molecular markers (Blasco-Costa et al., 2016a). Molecular approaches now represent the preferred method for resolving or confirming helminth life cycles (Blasco-Costa et al., 2016a; Blasco-Costa and Poulin, 2017; Faltýnková et al., 2022) and have been successfully applied to reconstruct trematode life cycles both among sympatric hosts (e.g., Soldánová et al., 2017; Assis et al., 2019) and across ecosystems (e.g., Locke et al., 2011; Blasco-Costa et al., 2016b; Bennett et al., 2023).

Trematode research in southern Africa has a long history (e.g., Cawston, 1917, 1921; Faust, 1920, 1921; Porter, 1938; Khalil and Polling, 1997; Scholz et al., 2016), and recent decades have seen substantial increase in comprehensive ecological and wildlife parasitological studies. As a result, a growing body of literature now integrates morphological and molecular identification of first and second intermediate host stages in the region (e.g., Hoogendoorn et al., 2019, 2020; Vermaak et al., 2021, 2023; Molaba et al., 2023; Truter et al., 2023, 2025; Outa and Avenant-Oldewage, 2024a, 2024b), complemented by fewer records from definitive hosts (e.g., King et al., 2018; Dos Santos et al., 2021; Truter et al., 2023). While these studies advance the understanding of southern African trematode diversity and contribute to reference databases required for comparing newly generated morphological and molecular data, only a few studies have integrated multiple developmental stages for life cycle reconstruction (e.g., King and Van As, 2000; Sirgel et al., 2012; Achatz et al., 2022; Louvard et al., 2025; Yong et al., 2025; Donough et al., 2026). Consequently, the majority of southern African trematode species remain known from single developmental stages, with their distinct life cycles only partially understood.

Given southern Africa's rich but incompletely documented trematode fauna, targeted efforts to not only identify but connect developmental stages across hosts are essential. The aim of this study was to investigate trematode infections in molluscs and fishes and to genetically link their developmental stages in order to partially reconstruct previously unresolved digenean trematode life cycles. In addition, assignment to species followed “open nomenclature” (ON) (see Sigovini et al., 2016) to facilitate and advance taxonomic stability and harmony where species identification is not completely possible due to uncertainty in the identity of all hosts in the completion of a species' life cycle. For example, in the current study the definitive hosts are unknown and species identification is highly reliant on the morphology of sexual adults.

## 2 Methods

### 2.1 Host sampling and morphological analyses

Snail and fish hosts were collected at multiple sampling sites across southern Africa between 2019 and 2025 (Table 1).

**Table 1:** Sampling localities of snail and fish hosts in South Africa (SA) and Zambia (ZA).

	n	Locality	Coordinates	Date
Snail hosts				
*Bulinus* sp.	40	Klein Letaba River, SA	23° 18′ 58.06″ S, 30° 41′ 22.44″ E	Autumn 2023
	44	Krom Rivier; SA	33° 56′ 34.91″ S, 24° 19′ 03.69″ E	Autumn 2024
	88	Magic Dam, SA	23° 05′ 20.71″ S, 30° 23′ 01.68″ E	Spring 2024
	5	Nandoni Dam, SA	22° 58′ 20.43″ S, 30° 34′ 44.64″ E	Spring 2024
	68	Boons, SA	26° 00′ 30.90″ S, 27° 13′ 55.80″ E	Spring 2025
Fish hosts				
*Clarias gariepinus*	17	Zambezi River (Barotse floodplain), ZA	15° 12′ 01.59″ S, 22° 58′ 09.27″ E	2019
*Enteromius oraniensis*	10	Seekoeivlei, SA	27° 41′ 01.00″ S, 29° 34′ 41.00″ E	2022

*Bulinus* spp. snails were collected using hand nets, sieves or by hand-picking from sediment, stones, and aquatic vegetation from the waterbodies and subsequently transported to the laboratory. Individual snails were placed in separate beakers containing water from their respective sampling sites and exposed to continuous light for three consecutive days to induce emergence of cercariae from infected hosts. Beakers were examined daily using a Zeiss Stemi 305 stereomicroscope equipped with a Zeiss Axiocam 208 camera (Carl Zeiss Microscopy GmbH, Germany) for the presence of cercariae in the water column. After the three-day period, all snails were dissected and screened for prepatent infections (parthenitae). Cercarial morphology was studied and documented using an Olympus BX-53 compound microscope with an Olympus SC50 digital microscope camera (Olympus Corp, Tokyo, Japan). Prior to dissection, snails were morphologically identified to the lowest possible taxonomic level, and their shell morphology was documented using a stereomicroscope with camera (Brown, 1994; Appleton, 2002). Representative individuals were preserved in 96 % ethanol for molecular confirmation of morphological identification.

Sampling of fish hosts was conducted in Zambia in 2019 for *Clarias gariepinus* (Burchell, 1822), and in South Africa in 2022 for *Enteromius oraniensis* (Barnard, 1943) (morphologically identified as *Enteromius anoplus* but revalidated following the recent revision of this genus by Kambikambi et al., 2021). Sampling permission was granted by the Ministry of Fisheries and Livestock (Department of Fisheries, Mongu, Zambia) and World Wide Fund for Nature for *C. gariepinus* and a research permit from the Department of Economic, Small Business Development, Tourism and Environmental Affairs (DESTEA), permit number 202107000007476 for *E. oraniensis*. Ethical clearance for the use and handling of target host species was provided by the AnimCare Ethics Committee of the North-West University (NWU), project ethics numbers NWU-00159-18-A5 and NWU-00781-22-A5. Sampling methods involved rod and reel, baited longlines, and electrofishing. Live fish were humanely killed via cranial pithing followed by cervical transection. Fish dissection and parasitological screening of the brain, cranial cavity, eyes, gills, musculature, and visceral tissue for trematodes followed the protocols described in Scholz et al. (2018a, 2018b). Encysted or encapsulated metacercariae were excysted using fine insect pins. Excysted and free metacercariae were subsequently rinsed in 0.9 % saline, heat fixed in saline and preserved as described below. Metacercarial morphology was studied using a Nikon Eclipse N*i* compound microscope (Nikon Instruments, Tokyo, Japan) equipped with a NIS-Elements BR 4.60 digital imaging system.

Preliminary identification of trematode developmental stages to family or genus level was based on morphological characteristics. Measurements were obtained from photomicrographs of live cercariae and fixed metacercariae using ImageJ image analysis software (Schneider et al., 2012) and are presented in micrometres (μm), with ranges followed by means in parentheses (length × width). Representative photomicrographs were used as photohologenophores (Pleijel et al., 2008). All individuals were stored in 96 % molecular grade ethanol for subsequent molecular analyses.

### 2.2 Molecular sequencing

DNA extraction was performed using the PCRBio Rapid Extract Lysis Kit (PCRBiosystems, Analytical Solutions, Randburg, South Africa) as described in Truter et al. (2025), with the final reaction dilution adjusted to 200 μL PCR grade water for metacercariae and 450 μL for snail, parthenitae and cercarial tissue. Molecular sequence data for snail hosts were generated for the cytochrome *c* oxidase I subunit (*cox*1) barcoding region (Table 2). Molecular sequence data for trematodes were generated for a section of the large ribosomal subunit (28S rDNA), the ITS1–5.8S–ITS2 internal transcribed spacer region (ITS1–2), and a section of *cox*1 and the nicotinamide adenine dinucleotide dehydrogenase subunit 1 (*nad*1) mitochondrial DNA (Table 2). All PCR reactions were carried out as 25 μL reaction volumes with 12.5 μL DreamTaq™ Hot Start Green PCR Master Mix, 1.25 μL of each primer (10 μM), 7 μL molecular-grade water, and 3 μL DNA. Thermocycling conditions for 28S rDNA, ITS1–2, and *cox*1 mtDNA were adjusted as given in Truter et al. (2025). Thermocycling conditions for *nad*1 mtDNA were adjusted as follows: initial denaturation at 95 °C for 5 min, 35 cycles of amplification at 94 °C for 30 s, 48 °C for 30 s, 72 °C for 45 s, and final extension at 72 °C for 7 min. The PCR products were purified and Sanger sequenced bidirectionally [Inqaba Biotechnical Industries (Pty) Ltd, Pretoria, South Africa] using the respective PCR primers and internal sequencing primers for the 28S rDNA region (Table 2).

**Table 2:** PCR and sequencing primers for gene fragments amplified in the present study.

Gene fragment	Primer name	Fragment length	Nucleotide sequence (5′–3′)	Reference
*cox*1 (snails)	LCO1490	∼660 base pairs (bp)	GGT CAA CAA AT C ATA AAG ATA TTG G	Folmer et al. (1994)
	HCO2198		TAA ACT TCA GGG TGA CCA AAA AAT CA	
28S	digl2	∼1200 bp	AAG CAT ATC ACT AAG CGG	Tkach et al. (2001)
	1500R		GCT ATC CTG AGG GAA ACT TCG	Snyder and Tkach (2001)
	300F∗		CAA GTA CCG TGA GGG AAA GTT G	Littlewood et al. (2000)
	ECD2∗		CTT GGT CCG TGT TTC AAG ACG GG	Tkach et al. (2003)
ITS1–5.8S–ITS2	D1	∼1000 bp	AGG AAT TCC TGG TAA GTG CAA G	Galazzo et al. (2002)
	D2		CGT TAC TGA GG GAA TCC TGG T	
*cox*1	Dice1F	∼820 bp	ATT AAC CCT CAC TAA ATT WCN TTR GAT CAT AAG	Van Steenkiste et al. (2015)
	Dice14R		TAA TAC GAC TCA CTA TAC CHA CMR TAA ACA TAT GAT G	
*nad*1	NDJ11	∼520 bp	AGA TTC GTA AGG GGC CTA ATA	Kostadinova et al. (2003)
	NDJ2a		CTT CAG CCT CAG CAT AAT	

∗Internal primers used for sequencing only.

### 2.3 Phylogenetic analyses

Newly generated sequences were assembled, edited and trimmed using Geneious Prime 2026.0.1 (https://www.geneious.com). Mitochondrial *cox*1 and *nad*1 sequences were checked for the amplification of pseudogenes using the trematode mitochondrial code (Translation Table 21; https://www.ncbi.nlm.nih.gov/taxonomy/utils/wprintgc.cgi#SG21) (Garey and Wolstenholme, 1989; Ohama et al., 1990). All sequences were then compared to the NCBI database using a BLASTn search to identify congeners for inclusion in the phylogenetic analyses. Sequences from GenBank with close similarity to the new isolates were aligned with sequences from the present study using MUSCLE 5.1 algorithm (Edgar, 2004) implemented in Geneious Prime. In total, seven alignments were generated: Alignment 1 (Echinostomatidae, 28S rDNA, 53 sequences); Alignment 2 (Echinostomatidae, ITS1–2, 29 sequences); Alignment 3 (Echinostomatidae, *cox*1, 27 sequences); Alignment 4 (Echinostomatidae, *nad*1, 16 sequences); Alignment 5 (Diplostomidae, 28S rDNA, 21 sequences), Alignment 6 (Diplostomidae, ITS1–2, 38 sequences), Alignment 7 (Diplostomidae, *cox*1, 31 sequences) (Table S1). All alignments were trimmed to the shortest sequence length and genetic divergence (uncorrected *p*-distances) calculated using MEGA v. 11 (Tamura et al., 2021) (Table S3–S9). Phylogenetic trees were generated using Maximum Likelihood (ML) and Bayesian Inference (BI). The best-fitting nucleotide substitution models were selected based on the Akaike Information Criterion (AIC) in ModelFinder (Kalyaanamoorthy et al., 2017) for ML and jModelTest2 v. 2.1.10 (Guindon and Gascuel, 2003; Darriba et al., 2012) for BI analyses (Table S1). The ML analyses were performed in IQ-TREE v. 2.4.0 (Minh et al., 2020) with 1000 bootstrap pseudoreplicates. The BI analyses were performed in MrBayes v. 2.3.7 (Ronquist et al., 2012) with 10,000,000 Markov chain Monte Carlo generations sampled every 1000 generations and only the final 75 % of trees used to produce the consensus. All trees were visualized and edited using FigTree v. 1.4.4 (http://tree.bio.ed.ac.uk/software/figtree/).

## 3 Results

Molecular sequencing of snails revealed that the collected material comprised several *Bulinus* species (Fig. S1). As species-level assignments based solely on shell morphology are often unreliable (Chibwana et al., 2020; Tumwebaze et al., 2022), and only a subset of snails was molecularly spot-checked, all snails from the present study were assigned to *Bulinus* sp.

Across the sampled hosts, data generated in the present study allowed for partial life cycle reconstruction of two trematode taxa.

### 3.1 *Petasiger* sp. 5 (Echinostomatidae)

First intermediate host: *Bulinus* sp. (Gastropoda: Planorbidae).

Locality and prevalence: Klein Letaba River, Mpumalanga, South Africa (2.5 %); Krom Rivier, Western Cape, South Africa (2.3 %); Magic Dam, Mpumalanga, South Africa (5.7 %); Nandoni Dam, Mpumalanga, South Africa (20.0 %); Boons, Gauteng, South Africa (2.9 %).

Second intermediate host: *Enteromius oraniensis* (Barnard, 1943) (Cypriniformes: Cyprinidae).

Locality and prevalence: Seekoeivlei, Free State, South Africa (10.0 %).

Site of infection: Gill filament (encysted).

Representative DNA sequence data: 28S rDNA — 1204 bp (GenBank accession numbers PX758998–PX758999, PX759001–PX759002, PX759004), ITS1–2 — 1211 bp (PX759006), *cox*1 mtDNA — 721 bp (PX775911–PX775912, PX775914–PX775918, PX775920), and *nad*1 mtDNA — 484 bp (PX763560).

#### 3.1.1 Molecular identification

**Fig. 1:**
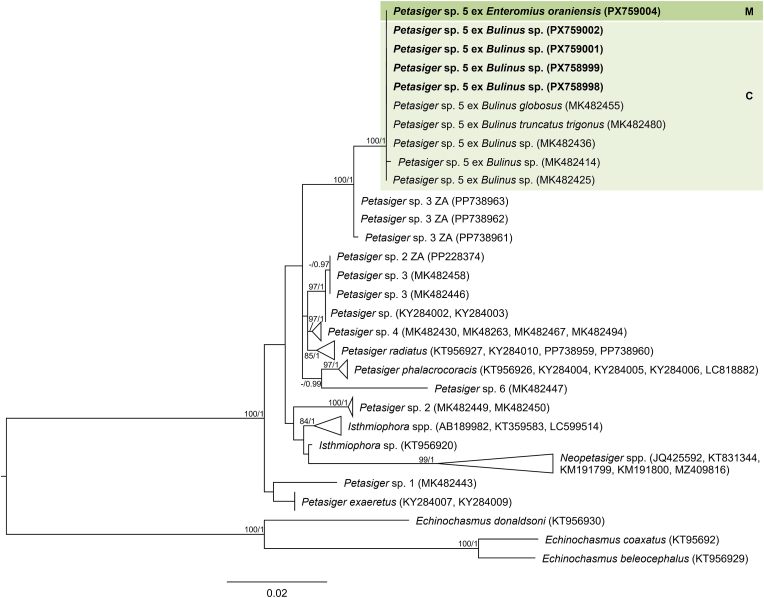
Maximum Likelihood (ML) phylogram of selected 28S rDNA sequences of family Echinostomatidae. Outgroups: *Echinochasmus beleocephalus, Echinochasmus coaxatus, Echinochasmus donaldsoni* (Echinochasmidae). Nodal support is given as bootstrap values (>70) for ML analyses and posterior probabilities (>0.90) for Bayesian Inference (BI) analyses. Host and life cycle stages (C, cercaria; M, metacercaria) are indicated for *Petasiger* sp. 5 (shaded). Sequences generated in this study are presented in bold.

**Fig. 2:**
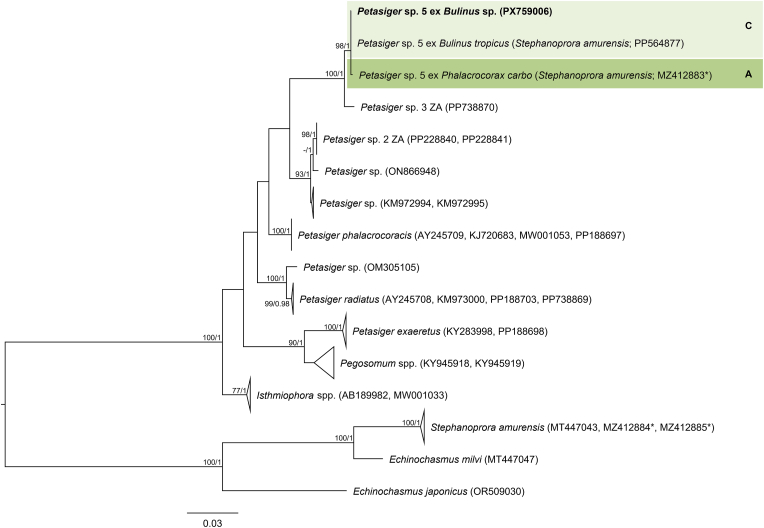
Maximum Likelihood (ML) phylogram of selected ITS1–2 sequences of family Echinostomatidae. Outgroups: *Echinochasmus japonicus, Echinochasmus milvi*, *Stephanoprora amurensis* (Echinochasmidae). Nodal support is given as bootstrap values (>70) for ML analyses and posterior probabilities (>0.90) for Bayesian Inference (BI) analyses. Host and life cycle stages (C, cercaria; A, adult) are indicated for *Petasiger* sp. 5 (shaded). Sequences generated in this study are presented in bold. Sequences marked with an asterisk (∗) indicate isolates from Chibwana and Katandukila (2021) that showed incongruent placement between ITS1–2 and *cox*1 phylogenies.

**Fig. 3:**
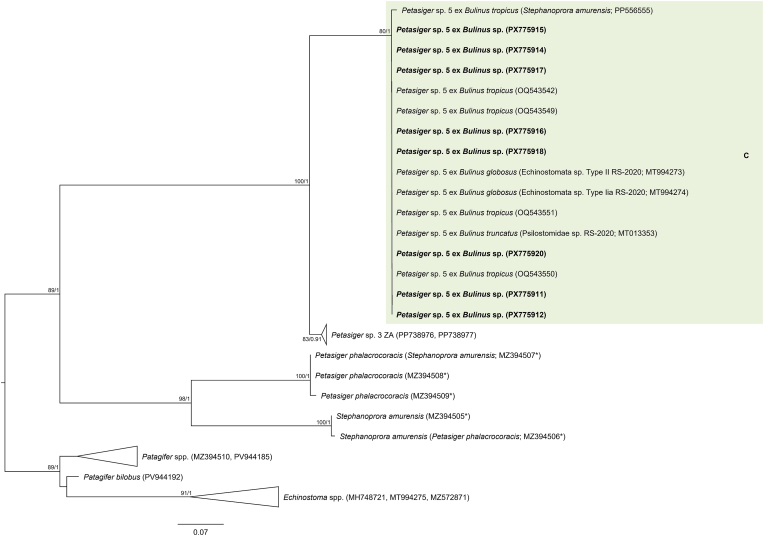
Maximum Likelihood (ML) phylogram of selected *cox*1 mtDNA sequences of *Petasiger* spp. Outgroups: *Echinostoma* spp., *Patagifer* spp. (Echinostomatidae). Nodal support is given as bootstrap values (>70) for ML analyses and posterior probabilities (>0.90) for Bayesian Inference (BI) analyses. Host and life cycle stage (C, cercaria) are indicated for *Petasiger* sp. 5 (shaded). Sequences generated in this study are presented in bold. Sequences marked with an asterisk (∗) indicate isolates from Chibwana and Katandukila (2021) that showed incongruent placement between ITS1–2 and *cox*1 phylogenies.

The final trimmed 28S alignment comprised 1143 bp. The newly generated 28S sequences from cercariae in *Bulinus* sp. and metacercariae from the gills of *E. oraniensis* were identical and differed by 0–0.09 % (0–1 bp) from isolates designated as *Petasiger* sp. 5 by Laidemitt et al. (2019) from *Bulinus* spp. in Kenya (Fig. 1, Table S3). ITS1–2, *cox*1 and *nad*1 data were obtained from cercarial isolates only. The trimmed ITS1–2 alignment comprised 1015 bp. The newly generated ITS1–2 sequence differed by 0.10 % (1 bp) from a sequence reported by Chibwana and Katandukila (2021) as *Petasiger phalacrocoracis* (Yamaguti, 1939) Skrjabin and Bashkirova, 1956 from the great cormorant *Phalacrocorax carbo* (Linnaeus, 1758), deposited in GenBank as *Stephanoprora amurensis* (Tatonova, Izrailskaia & Besprozvannykh, 2020) (MZ412883) (Fig. 2, Table S4). Based on the observed intraspecific divergence of 0–1 bp for this fragment among other *Petasiger* spp. (Table S4), these isolates can be considered conspecific. The trimmed *nad*1 alignment comprised 442 bp. The newly generated *nad*1 sequence differed by 0–0.45 % (0–2 bp) from *Petasiger* sp. 5 isolates reported by Laidemitt et al. (2019) and Hammoud et al. (2022) (Fig. S2, Table S6). The trimmed *cox*1 alignment comprised 407 bp. Isolates from the present study differed by 0–0.80 % (0–3 bp) from each other and by 0–1.34 % (0–5 bp) from *Petasiger* sp. 5 ex *Bulinus tropicus* (Krauss, 1848) from Uganda (Hammoud et al., 2025) (Fig. 3, Table S5). Based on this, the isolates from the present study were identified as *Petasiger* sp. 5. *Cox*1 isolates recovered from *Bulinus* spp. in Zimbabwe and deposited as ‘Echinostomata sp.’ (MT994273–274), ‘Psilostomidae sp.’ (MT013353), and *S. amurensis* (PP556555) (Schols et al., 2020; Mudavanhu et al., 2024) also fell within the range of intraspecific variation to the present study's isolates (0–1.12 % and 0–4 bp), and likely represent misidentified isolates of *Petasiger* sp. 5. ML and BI analyses of the nuclear 28S and ITS1–2 datasets yielded congruent topologies, recovering *Petasiger* sp. 5 as a strongly supported sister clade to isolates designated as *Petasiger* sp. 3 ZA from *Burnupia transvaalensis* (Craven, 1880) from South Africa (Outa and Avenant-Oldewage, 2024a) (Fig. 1, Fig. 2). Mitochondrial *nad*1 and *cox*1 phylogenies likewise grouped isolates from the present study with previously published *Petasiger* sp. 5 sequences, including the isolates of Schols et al. (2020) and Mudavanhu et al. (2024) in the *cox*1 phylogeny (Fig. 3, Fig. S2). In contrast to the ITS1–2 sequence reported by Chibwana and Katandukila (2021), the corresponding *cox*1 sequences did not group with *Petasiger* sp. 5, and instead formed a strongly supported monophyletic sister clade to sequences deposited as *S. amurensis* from the same study (Chibwana and Katandukila, 2021) (Fig. 3).

#### 3.1.2 Cercarial morphology

**Fig. 4:**
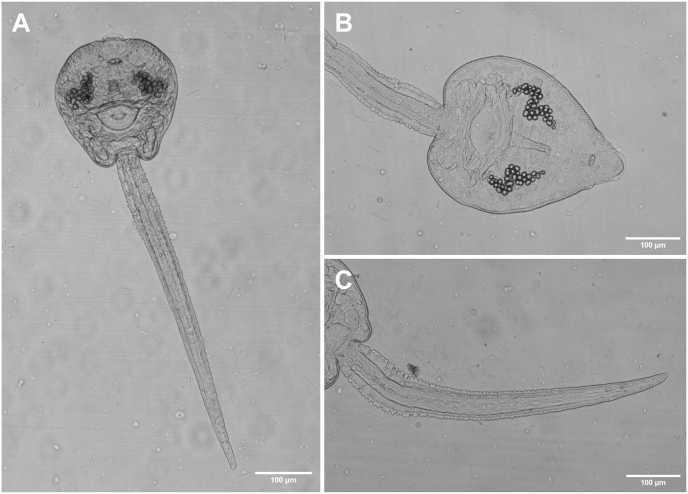
Photomicrographs of live cercariae of *Petasiger* sp. 5 ex *Bulinus* sp. [photohologenophore, GenBank accession numbers PX759001 (28S), PX775915 (*cox*1)]. **A** Body and tail, ventral view. **B** Body, ventral view. **C** Tail, ventral view.

Based on ten live specimens (Fig. 4). Body elongate oval, maximum width anterior to ventral sucker, 243–350 × 201–306 (301 × 254). Tail simple 493–641 (570) long, 1.8–2.2 (2.0) times longer than body, 32–45 (37) wide at base (Fig. 4C). Body surface covered with fine, posteriorly directed spines. Collar with 27 collar spines. Oral and ventral sucker surrounded by tegumental membranous rim. Oral sucker well-developed, subspherical, smaller than ventral sucker, 41–53 × 42–70 (46 × 53) (VS:OS length ratio 1.2:1, width ratio 1.3:1). Prepharynx present, pharynx ovoid, 19–22 × 17–21 (22 × 18), oesophagus well-pronounced, muscular, 43–70 (61) long, bifurcates into caeca at anterior margin of ventral sucker (Fig. 4B). Caeca reach to posterior end of body. Ventral sucker post-equatorial, transversely oval, 43–58 × 55–73 (53 × 67). Penetration gland cells indistinct, appear to be 3 pairs along oesophagus. Excretory ducts with 22–30 (26) granules, extending from excretory bladder to pharyngeal region (Fig. 4B). Cystogenous glands from posterior margin of oral sucker to posterior end of body. Flame cell pattern not observed.

#### 3.1.3 Metacercarial morphology

**Fig. 5:**
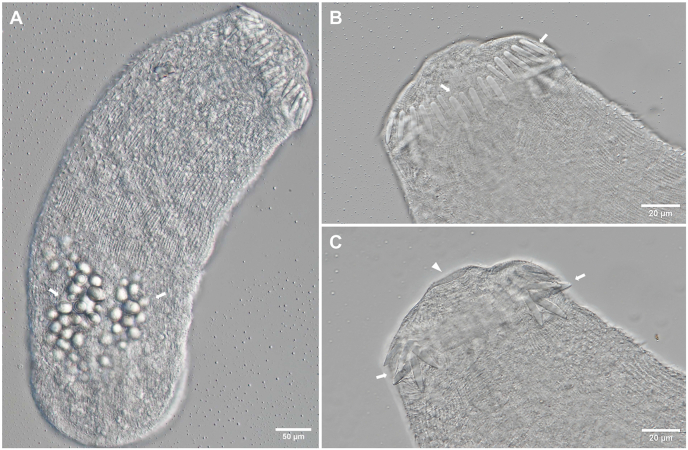
Photomicrographs of fixed metacercariae of *Petasiger* sp. 5 ex *Enteromius oraniensis* (Barnard, 1943) [photohologenophore, GenBank accession number PX759004 (28S)]. **A** Whole body, with excretory granules present (arrows). **B** Head collar, dorsal view, with dorsal collar spines (arrows). **C** Head collar with terminal oral sucker opening (arrowhead), dorsal view, with grouped collar spines (arrows).

Based on one excysted specimen (Fig. 5). Body 328 × 120, width measured at widest part. Prominent round excretory granules present in posterior third of body. Body tegument covered with fine, posteriorly directed spines (Fig. 5A). Collar with 27 circumoral spines. Spines robust, straight, 14–20 (17) long, groupings of 4 angle spines, 21–24 (22) (Fig. 5B and C). Oral sucker opening terminal (Fig. 5C). Prepharynx, pharynx, oesophagus, intestinal caeca and ventral sucker not observed.

### 3.2 *Tylodelphys* sp. 2 (Diplostomidae)

First intermediate host: *Bulinus* sp. (Gastropoda: Planorbidae).

Locality and prevalence: Klein Letaba River, Mpumalanga, South Africa (2.5 %).

Second intermediate host: *Clarias gariepinus* (Burchell, 1822) (Siluriformes: Clariidae).

Locality and prevalence: Barotse floodplain, Zambezi River, Western Province, Zambia (47.0 %).

Site of infection: Cranial cavity (free).

Representative DNA sequence data: 28S rDNA — 1164 bp (GenBank accession numbers PX759000, PX759003), ITS1–2 — 1247 bp (PX759005, PX759007), and *cox*1 mtDNA — 631 bp (PX775913, PX775919).

#### 3.2.1 Molecular identification

**Fig. 6:**
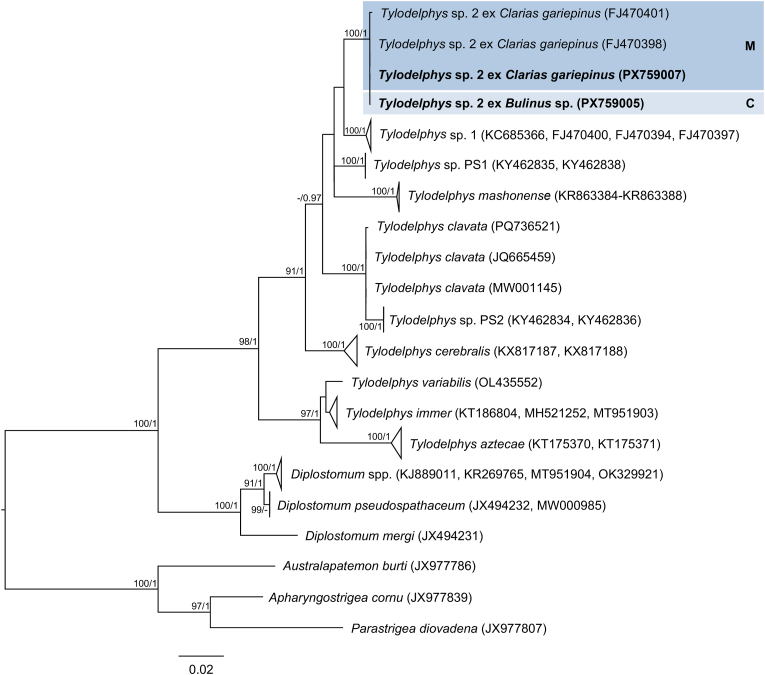
Maximum Likelihood (ML) phylogram of selected ITS1–2 sequences of family Diplostomidae. Outgroups: *Apharyngostrigea cornu, Australapatemon burti, Parastrigea diovadena* (Strigeidae). Nodal support is given as bootstrap values (>70) for ML analyses and posterior probabilities (>0.90) for Bayesian Inference (BI) analyses. Host and life cycle stages (C, cercaria; M, metacercaria) are indicated for *Tylodelphys* sp. 2 (shaded). Sequences generated in this study are presented in bold.

**Fig. 7:**
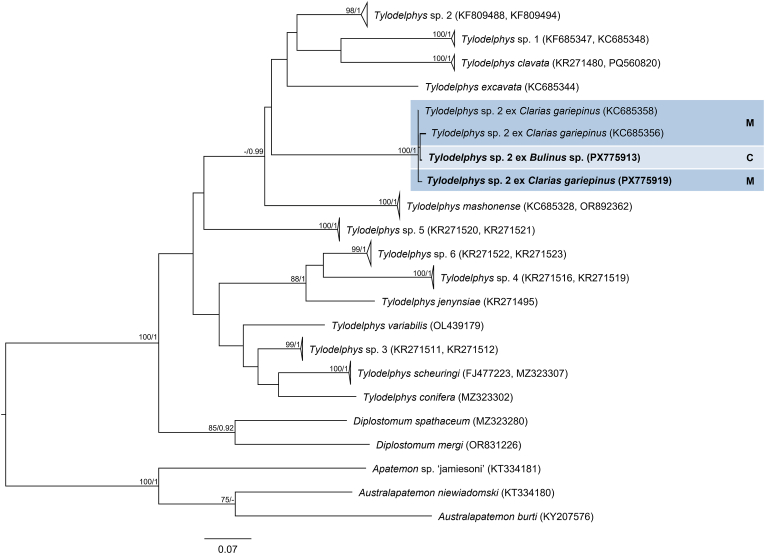
Maximum Likelihood (ML) phylogram of selected *cox*1 mtDNA sequences of family Diplostomidae. Outgroups: *Apatemon* sp. ‘jamiesoni’, *Australapatemon burti*, *Austrolapatemon niewiadomski* (Strigeidae). Nodal support is given as bootstrap values (>70) for ML analyses and posterior probabilities (>0.90) for Bayesian Inference (BI) analyses. Host and life cycle stages (C, cercaria; M, metacercaria) are indicated for *Tylodelphys* sp. 2 (shaded). Sequences generated in this study are presented in bold.

The final 28S alignment comprised 1097 bp. Newly generated 28S sequences obtained from cercariae from *Bulinus* sp. and metacercariae from the cranial cavity of *C. gariepinus* were identical. No conspecific 28S reference sequences were available in GenBank. The closest available sequence, *Tylodelphys mashonense* (Beverley-Burton, 1962) reported by Moema et al. (2013), differed by 0.91 % (10 bp) (Fig. S3, Table S7). The trimmed ITS1–2 alignment comprised 980 bp. The newly generated sequences were identical and differed by 0–0.11 % (0–1 bp) from isolates designated as *Tylodelphys* sp. 2 from *C. gariepinus* in Tanzania (Chibwana et al., unpublished) (Fig. 6, Table S8). The trimmed alignment length for *cox*1 sequences was 420 bp. The newly generated isolates differed by 0.48 % (2 bp) from each other and by 0.48–1.40 % (2–6 bp) from isolates of *Tylodelphys* sp. 2 of *C. gariepinus* in Tanzania (Chibwana et al., 2013) (Fig. 7, Table S9). Based on this data, isolates from the present study were identified as *Tylodelphys* sp. 2. Both ML and BI analyses of the ITS1–2 alignment recovered *Tylodelphys* sp. 2 as a strongly supported clade (Fig. 6). Likewise, ML and BI analyses of the *cox*1 dataset were congruent and placed *Tylodelphys* sp. 2 in a well-supported clade that includes cercariae from *Bulinus* sp. with both novel and previously published metacercariae from *C. gariepinus*, confirming the conspecificity of these developmental stages (Fig. 7).

#### 3.2.2 Cercarial morphology

**Fig. 8:**
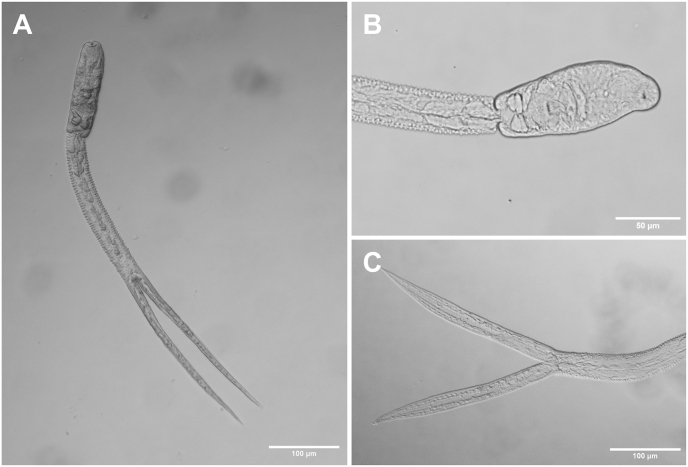
Photomicrographs of live cercariae of *Tylodelphys* sp. 2 ex *Bulinus* sp. [photohologenophore, GenBank accession numbers PX759000 (28S), PX759005 (ITS1–2), PX775913 (*cox*1)]. **A** Body and tail, ventral view. **B** Body, ventral view. **C** Tail and furcae, ventral view.

Based on three live specimens (Fig. 8). Body elongate, maximum width at level of ventral sucker, 117–139 × 36–49 (128 × 44). Tail 487–498 (492) long, 3.5–4.2 (3.9) times longer than body (Fig. 8A). Tail stem 228–240 × 26–33 (234 × 30). Furcae laterally flattened, 257–267 (262) long. Tail without fin fold. Longitudinal furrow extends from base and terminates near tips of furcae (Fig. 8C). Anterior end of body covered with minute, posteriorly directed spines. Oral sucker subspherical, larger than ventral sucker, 20–24 × 23–25 (22 × 24) (VS:OS length ratio 1:1.8, width ratio 1:1.6). Pharynx indistinct. Oesophagus short, bifurcates into well-developed caeca, extending to posterior end of body. Ventral sucker subspherical, small, 11–12 × 14–16 (12 × 15), with 27 spines at outer margin. Penetration gland cells 4, large, round to oval, anterior to ventral sucker (Fig. 8B). Excretory bladder small, triangular. Flame cell pattern not observed.

#### 3.2.3 Metacercarial morphology

**Fig. 9:**
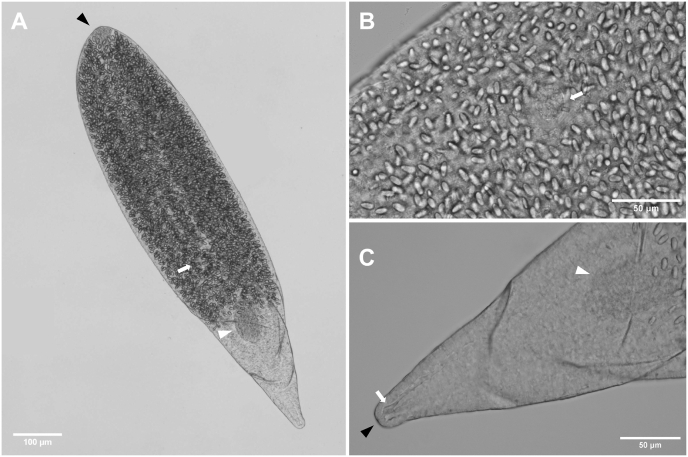
Photomicrographs of fixed metacercariae of *Tylodelphys* sp. 2 ex *Clarias gariepinus* (Burchell, 1822) [photohologenophore, GenBank accession numbers PX759003 (28S), PX759007 (ITS1–2), PX775919 (*cox*1)]. **A** Whole body, ventral view, oral sucker (black arrowhead), approximate position of ventral sucker (arrow), holdfast organ (white arrowhead). **B** Ventral sucker (arrow), ventral view. **C** Posterior end, ventral view, holdfast organ (white arrowhead), excretory bladder (arrow), excretroy pore (black arrowhead).

Based on one specimen (Fig. 9). Body elongate lacrimiform, anterior end rounded, posterior end conical with rounded end; length 928, width at widest part of body 211. Tegument without spines. Hindbody not well differentiated, 159 long (17 % of total body length), 101 wide at widest anterior margin. Oral sucker subterminal, subspherical, larger than ventral sucker, 60 × 52 (VS:OS length ratio 1:2.1, width ratio 1:2.7) (Fig. 9A). Pseudosuckers absent. Prepharynx, pharynx, oesophagus not observed. Ventral sucker small, subspherical, 22 × 25, distance from oral sucker 466 (50 % of body) (Fig. 9B). Intestinal caeca not observed, obscured by dense presence of oval granular inclusions (Fig. 9A). Holdfast organ muscular and oval, 95 × 63, distance from ventral sucker 142 (15 % of body) (Fig. 9C). Excretory bladder V-shaped, excretory pore terminal position at posterior tip of body (Fig. 9C).

## 4 Discussion

The present study presents the first record of previously unknown developmental stages of *Petasiger* sp. 5 and *Tylodelphys* sp. 2 and links their first and second intermediate hosts through molecular matching. The findings from the present study thus allow for partial life cycle reconstruction of both taxa and expand current knowledge of trematode-host associations in African freshwater ecosystems.

For *Petasiger* sp. 5, molecular data confirmed the conspecificity between cercariae from *Bulinus* sp. and metacercariae encysted in the gills of *E. oraniensis*. First intermediate host infections of this lineage have previously been reported from several *Bulinus* spp. in Kenya (Laidemitt et al., 2019) as well as from *B. tropicus* in Uganda (Hammoud et al., 2022), indicating that planorbid snails of the genus *Bulinus* (O. F. Müller, 1781) serve as suitable first intermediate hosts. The *cox*1 phylogeny generated in the present study is consistent with the data reported by Outa and Avenant-Oldewage (2024a), and further suggest that *Petasiger* sp. 5 has been recorded but misidentified from *Bulinus globosus* (Morelet, 1866) (MT994273, reported as ‘Echinostomata sp.’), *Bulinus truncatus* (Audouin, 1827) (MT013353, reported as ‘Psilostomidae sp.’) and *B. tropicus* (PP556555, reported as ‘*Stephanoprora amurensis*’) in Zimbabwe (Schols et al., 2020; Mudavanhu et al., 2024). By identifying *E. oraniensis* as the second intermediate host, the present study provides the first confirmed record of *Petasiger* sp. 5 metacercariae infecting cyprinids of the Smiliogastrinae in Africa. The newly generated ITS1–2 data further suggest that an isolate of adult *Petasiger* sp. 5 has been recorded as *P. phalacrocoracis* from *Phalacrocorax carbo* (MZ412883, ‘*Stephanoprora amurensis*’) in Tanzania by Chibwana and Katandukila (2021). However, uncertainty remains as the corresponding *cox*1 sequences (MZ394507–MZ394509) from their study did not cluster with *cox*1 sequences of *Petasiger* sp. 5 from the present or previous studies (Fig. 3). Given the discrepancy between these genetic markers, the published sequences of Chibwana and Katandukila (2021) require re-evaluation. Future records of adult *Petasiger* spp., compared against the expanding reference database, will be necessary to confirm *P. carbo* as the definitive host of *Petasiger* sp. 5. Nonetheless, *P. carbo* represents a plausible definitive host, as *Petasiger* species typically use planorbid and lymnaeid snails as first intermediate hosts, cyprinid fishes and amphibians as second intermediate hosts, and birds of the families Phalacrocoracidae, Anhingidae, Ciconiidae, and Sulidae as definitive hosts (Tkach et al., 2016). Within sub-Saharan Africa, two nominal species [*Petasiger variospinosus* (Odhner, 1910) Yamaguti, 1933*,* and *Petasiger radiatus* (Dujardin, 1845) Tkach, Kudlai & Kostadinova, 2015] and several undescribed genetic lineages have been documented from first intermediate hosts (King and Van As, 2000; Laidemitt et al., 2019; Outa et al., 2020; Outa and Avenant-Oldewage, 2024a). In contrast, only three species (*P. radiatus, P. variospinosus*, *P. phalacrocoracis*) have been reported from definitive hosts (Bisseru, 1957; King and Van As, 2000; Chibwana and Katandukila, 2021), and, as described above, the latter requires validation through further molecular reference data (see also Outa and Avenant-Oldewage, 2024a). Among these, only *P. variospinosus* has a fully resolved life cycle, experimentally demonstrated using naturally infected *B*. *tropicus* to infect *Xenopus laevis laevis* (Daudin, 1802) tadpoles and subsequently the reed cormorant *Microcarbo africanus* (J. F. Gmelin, 1789) (King and Van As, 2000).

*Tylodelphys* sp. 2 has previously been recorded only as metacercariae infecting the cranial cavity and perineural adipose tissues of *C*. *gariepinus* in Tanzania (Musiba and Nkwengulila, 2006; Chibwana and Nkwengulila, 2010). The current study provides the first description of its cercarial stage and identifies *Bulinus* sp. as its first intermediate host. Species of *Tylodelphys* typically use lymnaeid or planorbid snails as first intermediate hosts, fishes and amphibians as second intermediate hosts, and piscivorous or amphibian-eating birds (e.g., Phalacrocoracidae, Ciconiidae, Sulidae) as definitive hosts (Blasco-Costa et al., 2017). In sub-Saharan Africa, the genus includes several nominal species [e.g., *Tylodelphys clavata* (von Nordmann, 1832) Diesing, 1850, *Tylodelphys grandis* (Zhokhov et al., 2010), *T. mashonense,* and *Tylodelphys xenopi* (Nigrelli and Maraventano, 1944)] (King and Van As, 1997; Zhokhov et al., 2010; Chibwana et al., 2015; Chibwana and Nkwengulila, 2016; Maraganga et al., 2024) as well as undescribed lineages recorded solely as metacercariae, e.g., *Tylodelphys* sp. 1–2 of Chibwana and Nkwengulila (2010) from *C. gariepinus* in Tanzania and *Tylodelphys* sp. 2 of (Otachi et al., 2015) from multiple freshwater fish hosts across several families in Kenya. Knowledge on African first intermediate hosts of *Tylodelphys* spp. remains particularly scarce, as prior to this study, cercarial stages were known only for *T. xenopi* from *B. tropicus* in South Africa and *T. mashonense* from *Bulinus* sp. in Tanzania (King and Van As, 1997; Chibwana et al., 2015). These species thus represent the only two with a fully resolved life cycle, in which *T. xenopi* infects *X. laevis laevis* and subsequently the Oriental darter *Anhinga melanogaster* (Pennant, 1769), whereas *T. mashonense* was matched to metacercariae in *C. gariepinus* and sexual adults in the grey heron *Ardea cinerea* (Linnaeus, 1758) and the great egret *Ardea alba* (Linnaeus, 1758) (King and Van As, 1997; Chibwana et al., 2015; Chibwana and Nkwengulila, 2016). Findings from the current study thus represent the third confirmed first intermediate host association for the genus *Tylodelphys* (Diesing, 1850) in Africa and provide an important step toward resolving the life cycle of *Tylodelphys* sp. 2.

The present study underscores the integration of molecular methods as the preferred approach for elucidating complex helminth life cycles (Locke et al., 2011; Chibwana et al., 2015; Selbach et al., 2015; Blasco-Costa and Poulin, 2017; Presswell and Blasco-Costa, 2020), as it has proved essential for matching trematode developmental stages in both taxa examined in this study due to limited morphological reference data. Under these circumstances, morphological matching alone would have remained tentative, as many diagnostic characters used for identification of trematode developmental stages are known to show high variability or substantial overlap among species. For the genus *Petasiger* (Dietz, 1909), Outa and Avenant-Oldewage (2024a) recently summarised that key cercarial traits frequently overlap among species and genetic lineages. Similarly, Laidemitt et al. (2019) listed the number of 19–20 refractory granules in each main excretory duct as a discriminating feature for *Petasiger* sp. 5, whereas cercariae studied in the present study contained 22–30 granules, a range shared with several other *Petasiger* species. The life cycle reconstruction in the present study further opposes the previously proposed conspecificity of *Petasiger* sp. 5 with *P. variospinosus* or *Cercaria decora* (Fain, 1953) based on morphological diagnostic features (Laidemitt et al., 2019), as *P. variospinosus* uses amphibians rather than fishes as second intermediate hosts (King and Van As, 2000). Comparable challenges occur among *Tylodelphys* spp., where Blasco-Costa et al. (2017) reviewed a higher number of nominal morphospecies than genetically validated lineages (Blasco-Costa et al., 2017 and references therein). Consequently, molecular matching remains essential for linking larval stages and reconstructing life cycles, provided that marker choice and reference data are carefully evaluated to avoid misidentification or incomplete matching (Blasco-Costa et al., 2016a). In parallel, it is essential that taxonomists follow a unified approach to the assignment of species names, even when all life stages are not yet known, using ON. Although ON is not formally recognised by the International Code of Zoological Nomenclature (ICZN), its consistent implementation remains critical in preserving taxonomic stability and preventing confusion regarding the true diversity of a genus when intermediate stages are discovered without knowledge of adult stages and the associated definitive host. The value of ON is well illustrated in diplostomid genera such as *Bolpbophorus* (Dubois, 1935), *Diplostomum* (von Nordmann, 1832), *Ornithodiplostomum* (Dubois, 1936), *Posthodiplostomum* (Dubois, 1936), and *Uvulifer* (Yamaguti, 1934), where it has provided a coherent framework for tracking putative species diversity, with sequential identifiers indicating the number of recognised lineages and facilitating comparison across studies (see Hoogendoorn et al., 2019, 2020 and references therein). In contrast, the random assignment of ‘identifiers’ (e.g., isolates, morphotypes, or ‘sp. A’ and ‘sp. B’) within individual or geographically isolated studies tends to create confusion. This is exemplified in representatives of the genus *Tylodelphys* from fish hosts in Tanzania, where the assignment to ‘sp. 1’, ‘sp. 2’, ‘sp. X’ and ‘sp. Y’ was applied to isolates recovered from the same host (*C. gariepinus*) and locality across different years. Inconsistent naming obscures the diversity and number of putative species with a given host and region, complicates assessments of co-infections through time, and makes it unclear whether the same putative species have been repeatedly reported under different identifiers. For the genus *Tylodelphys*, ‘sp. X’ and ‘sp. Y’ are in fact synonymous with ‘sp. 1’ and ‘sp. 2’, respectively (see Mwita and Nkwengulila, 2005; Musiba and Nkwengulila, 2006; Mwita and Nkwengulila, 2008, 2010; Mwita, 2011, 2014a, 2014b), and Chibwana et al. (2013) later utilised phylogenetic inferences to delimit ‘sp. 1’ and ‘sp. 2’ as separate species. Given the repeatedly documented incongruent metacercarial morphology within the genus (e.g., Chibwana et al., 2013; Blasco-Costa et al., 2017), this emphasizes caution when assigning species names solely based on intermediate stage morphology.

By documenting previously undescribed developmental stages of *Petasiger* sp. 5 and *Tylodelphys* sp. 2 and providing the genetic links for partial life cycle reconstruction, the present study enhances the understanding of diversity, host use and life cycle strategies of trematodes in African freshwater ecosystems. Formal descriptions of *Petasiger* sp. 5 and *Tylodelphys* sp. 2 still await the documentation of sexual adults from definitive hosts. However, with their first and second intermediate host stages identified, species descriptions can now integrate all developmental stages, as recommended for integrative trematode taxonomy (e.g., Blasco-Costa and Poulin, 2017; Presswell and Blasco-Costa, 2020; Cribb et al., 2025).

## CRediT authorship contribution statement

**Annabell Hüsken:** Writing – original draft, Visualization, Investigation, Formal analysis, Writing – review & editing. **Marliese Truter:** Writing – original draft, Visualization, Investigation, Conceptualization, Writing – review & editing. **Jessica Schwelm:** Writing – review & editing, Investigation, Conceptualization. **Bernd Sures:** Writing – review & editing, Supervision. **Nico J. Smit:** Writing – review & editing, Supervision, Resources. **Wynand Malherbe:** Writing – review & editing, Supervision, Resources.

## Ethical aspects

All procedures contributing to this work comply with the ethical standards of the relevant national and institutional guides on the care and use of animals. Ethical approval for the use of animals for research purposes was obtained from the North-West University AnimCare Research Ethics Committee (NWU-00159-18-A5; NWU-00781-22-A5). Temporary holding of fish and humane killing followed protocols stipulated in SOP NWU-00272-17-A5 and NWU-00267-17-A5. Research permits and/or permission for fish collection for the purpose of research were obtained prior to sampling from the relevant provincial departments and authorities.

## Financial support

This research is part of the larger REFRESH project funded by the Foundational Biodiversity Initiative Programme (FBIP) of the 10.13039/501100001321National Research Foundation of South Africa (REFRESH—FBIP-211006643719; Grant no. 138573). A. Hüsken was supported by a research grant from the DAAD-Stiftung (Treuhänder: DAAD e.V.). M. Truter was funded by the 10.13039/501100005274North-West University Postdoctoral fellowship programme and an NRF-South African Research Chairs Initiative, Inland Fisheries and Freshwater Ecology grant (Grant no. 110507). We also acknowledge the use of infrastructure and equipment provided by the NRF-SAIAB Research Platforms and the funding channelled through the NFR-SAIAB Institutional Support system. Opinions, findings, conclusions and recommendations expressed in this publication are that of the authors, and the NRF accepts no liability whatsoever in this regard.

## Declaration of competing interest

The authors declare that they have no known competing financial interests or personal relationships that could have appeared to influence the work reported in this paper.

## Data Availability

All data generated or analysed in this study are available within the article. Newly generated sequences have been deposited in GenBank and are available under accession numbers PX758998–PX759007, PX763560, PX775911–PX775920, and PX776006–PX776008.
