# Supplementary material for: Bridging knowledge gaps in two trematode life cycles: insights from southern African freshwater ecosystems
Source: Int J Parasitol Parasites Wildl. 2026 Feb 13;29:101205. doi: 10.1016/j.ijppaw.2026.101205 (PMC13083302; doi:10.1016/j.ijppaw.2026.101205)
Supplement: Multimedia component 1 [file mmc1.docx]

**Supplementary file 1.** Details on the sequence alignments and sequences used in the phylogenetic analyses of the current study (Tables S1–S2), and Maximum Likelihood phylograms of *Bulinus* spp. (Alignment 8, *cox*1), *Petasiger* spp. (Alignment 4, *nad*1), and Diplostomidae (Alignment 5, 28S) (Figs S1–S3).

**Table S1** Details on the sequence alignments generated in the present study.

| **Trematode family/taxa** | **Gene region** | **Alignment** | **No. of newly generated sequences** | **No. of GenBank sequences** | **Alignment length** | **Model ML** | **Model BI** | **Outgroup** |
| --- | --- | --- | --- | --- | --- | --- | --- | --- |
| Echinostomatidae | 28S | 1 | 5 | 48 | 1,143 | GTR+F+R2 | GTR+G | *Echinochasmus beleocephalus*,  *E. coaxatus*, *E. donaldsoni*  (Echinochasmidae) |
|  | ITS1–2 | 2 | 1 | 28 | 1,015 | TVM+F+G4 | GTR+G | *Echinochasmus japonicus,  E. milvis, Stephanoprora amurensis* (Echinochasmidae) |
|  | *cox*1 | 3 | 8 | 19 | 373 | TIM3+F+G4 | HKY+G | *Echinostoma* spp., *Patagifer* spp. (Echinostomatidae) |
|  | *nad*1 | 4 | 1 | 15 | 442 | TVM+F+G4 | GTR+G | *Drepanocephalus auratus* (Echinostomatidae) |
| Diplostomidae | 28S | 5 | 2 | 19 | 1,097 | GTR+F+R3 | GTR+G | *Apatemon gracilis, Apharyngostrigea cornu, Parastrigea diovadena* (Strigeidae) |
|  | ITS1–2 | 6 | 2 | 36 | 980 | TPM2u+F+R2 | GTR+G | *Apharyngostrigea cornu*, *Australapatemon burti*,  *Parastrigea diovadena*  (Strigeidae) |
|  | *cox*1 | 7 | 2 | 29 | 420 | GTR+F+I+R3 | GTR+G | *Apatemon* sp. ‘jamiesoni’, *Australapatemon* *burti*,  *Au.* *niewiadomski* (Strigeidae) |
| *Bulinus* spp.  (Gastropoda: Planorbidae) | *cox1* | 8 | 3 | 21 | 540 | GTR+F+G4 | GTR+G | *Indoplanorbis exustus* (Planorbidae) |

**Table S2** Summary of all sequences used for the phylogenetic analyses. Sequences generated in this study are highlighted in bold. Life cycle stages: A, sexual adult; S, sporocyst; R, redia; C, cercaria; M, metacercaria.

| **Taxon** | **Life cycle stage** | **Host** | **Location** | **GenBank Accession No.** | | | **Reference** |
| --- | --- | --- | --- | --- | --- | --- | --- |
|  |  |  |  | **28S** | **ITS1–5.8S–ITS2** | ***cox*1 / *nad*1*** |  |
| **Family Diplostomidae** | | | | | | | |
| *Diplostomum gavium* | – | *Lymnaea* sp. | USA | MZ314157 | – | – | Achatz et al. (2022b) |
| *Diplostomum mergi* | C | *Ampullaceana balthica* | Denmark | – | JX494231 | – | Haarder et al. (2013) |
| *Diplostomum mergi* | M | *Gobius niger* | Finland | – | – | OR831226 | Martinek and Hernández-Orts (2023) |
| *Diplostomum paracaudum* | M | *Gadus morhua* | Denmark | – | KJ889011 | – | Mehrdana et al. (2015) |
| *Diplostomum paracaudum* | M | *Oncorhynchus mykiss* | Denmark | – | OK329921 | – | Karami et al. (2022) |
| *Diplostomum phoxini* | M | *Phoxinus phoxinus* | Germany | MZ616380 | – | – | Schwelm et al. (2021) |
| *Diplostomum pseudospathaceum* | C | *Stagnicola palustris* | Denmark | – | MW000985 | – | Duan et al. (2021) |
| *Diplostomum pseudospathaceum* | C | *Ampullaceana balthica* | Denmark | – | JX494232 | – | Haarder et al. (2013) |
| *Diplostomum rauschi* | A | *Chroicocephalus genei* | Ukraine | MZ314169 | – | – | Achatz et al. (2022b) |
| *Diplostomum spathaceum* | C | *Larus ridibundus* | Czech Republic | – | KR269765 | – | Brabec et al. (2015) |
| *Diplostomum spathaceum* | M | *Coregonus lavaretus* | Russia | – | MT951904 | – | Ieshko et al. (2022) |
| *Diplostomum spathaceum* | A | *Larus cachinnans* | Ukraine | MZ314171 | – | MZ323280 | Achatz et al. (2022b) |
| *Tylodelphys aztecae* | A | *Podilymbus podiceps* | Mexico | MF398337 | – | – | Hernández-Mena et al. (2017) |
| *Tylodelphys aztecae* | A | *Podiceps nigricollis* | Mexico | MF624786 | – | – | López-Jiménez et al. (2018) |
| *Tylodelphys aztecae* | M | *Goodea atripinnis* | Mexico | – | KT175370–KT175371 | – | García-Varela et al. (2016) |
| *Tylodelphys cerebralis* | M | *Channa punctate* | India | – | KX817187–KX817188 | – | Chaudhary et al. (2017b) |
| *Tylodelphys clavata* | C | *Ampullaceana balthica* | Czech Republic | PQ738921 | PQ736521 | – | Kundid et al. (2025) |
| *Tylodelphys clavata* | C | *Ampullaceana balthica* | Denmark | – | MW001145 | – | Duan et al. (2021) |
| *Tylodelphys clavata* | M | *Coregonus lavaretus* | Germany | – | JQ665459 | – | Behrmann-Godel (2013) |
| *Tylodelphys clavata* | M | – | Italy | – | – | KR271480 | Locke et al. (2015) |
| *Tylodelphys clavata* | M | *Neogobius fluviatilis* | Lithuania | – | – | PQ560820 | Kudlai et al. (2024) |
| *Tylodelphys conifera* | A | *Podiceps grisegena* | USA | MZ314185 | – | MZ323302 | Achatz et al. (2022b) |
| *Tylodelphys excavata* | C | *Planorbarius corneus* | Czech Republic | – | – | KC685344 | Chibwana et al. (2015) |
| *Tylodelphys excavata* | M | *Pelophylax ridibundus* | Ukraine | OL435551 | – | – | Achatz et al. (2022a) |
| *Tylodelphys excavata* | C | *Planorbarius corneus* | Russia | OP714395 | – | – | Svinin et al. (2023) |
| *Tylodelphys jenynsiae* | M | *Cnesterodon decemmaculatus* | Argentina | – | – | KR271495 | Locke et al. (2015) |
| *Tylodelphys immer* | M | *Salvelinus fontinalis* | Canada | – | KT186804 | – | Locke et al. (2015) |
| *Tylodelphys immer* | M | *Coregonus lavaretus* | Russia | – | MT951903 | – | Ieshko et al. (2022) |
| *Tylodelphys immer* | A | *Gavia immer* | Canada | MH521252 | MH521252 | – | Locke et al. (2018) |
| *Tylodelphys mashonense* | M | *Tilapia sparrmanii* | South Africa | KF189071 | – | – | Moema et al. (2013) |
| *Tylodelphys mashonense* | M | *Clarias gariepinus* | Tanzania | – | KR863384–KR863388 | KC685328 | Chibwana et al. (2015) |
| *Tylodelphys mashonense* | M | *Clarias gariepinus* | Kenya | – | – | OR892362 | Maraganga et al. (2023) |
| *Tylodelphys scheuringi* | M | *Umbra limi* | USA | MZ314188 | – | MZ323307 | Achatz et al. (2022b) |
| *Tylodelphys scheuringi* | – | – | Canada | – | – | FJ477223 | Moszczynska et al. (2009) |
| *Tylodelphys* sp.  BU384 | S | *Radix auricularia* | Poland | OQ672262 | – | – | Kanarek et al. (2023) |
| *Tylodelphys* sp. 1  AK-2013 | M | *Clarias gariepinus* | Tanzania | – | KC685366 | KC685347–KC685348 | Chibwana et al. (2013) |
| *Tylodelphys* sp.  1 FC-2009 | M | *Clarias gariepinus* | Tanzania | – | FJ470394, FJ470397, FJ470400 | – | Chibwana et al. (unpublished) |
| *Tylodelphys* sp. 2  FC-2009 | M | *Clarias gariepinus* | Tanzania | – | FJ470398, FJ470401 | – | Chibwana et al. (unpublished) |
| *Tylodelphys* sp. 2  SAL-2014 | M | – | Kenya | – | – | KF809488, KF809494 | Otachi et al. (2015) |
| *Tylodelphys* sp. 2  AK-2013 | M | *Clarias gariepinus* | Tanzania | – | – | KC685356, KC685358 | Chibwana et al. (2013) |
| ***Tylodelphys* sp. 2 AH-2023** | **C** | ***Bulinus* sp.** | **South Africa** | **PX759000** | **PX759005** | **PX775913** | **This study** |
| ***Tylodelphys* sp. 2 AH-2019** | **M** | ***Clarias gariepinus*** | **Zambia** | **PX759003** | **PX759007** | **PX775919** | **This study** |
| *Tylodelphys* sp. 3  SAL-2014 | M | *Lepomis microlophus* | USA | – | – | KR271511–KR271512 | Locke et al. (2015) |
| *Tylodelphys* sp. 4  SAL-2014 | M | *Gobiomorus maculatus* | Mexico | – | – | KR271516, KR271519 | Locke et al. (2015) |
| *Tylodelphys* sp. 5  SAL-2014 | M | *Dormitator latifrons* | Mexico | – | – | KR271520–KR271521 | Locke et al. (2015) |
| *Tylodelphys* sp. 6  SAL-2014 | M | *P. latipinna* | USA | – | – | KR271522–KR271523 | Locke et al. (2015) |
| *Tylodelphys* sp.  PS1 AC-2017 | M | *Puntius sophore* | India | – | KY462835, KY462838 | – | Chaudhary et al. (2017a) |
| *Tylodelphys* sp.  PS2 AC-2017 | M | *Puntius sophore* | India | – | KY462834, KY462836 | – | Chaudhary et al. (2017a) |
| *Tylodelphys* sp.  VVT1 | – | *Ambystoma talpoideum* | USA | MZ314189 | – | – | Achatz et al. (2022b) |
| *Tylodelphys variabilis* | A | *Didelphis virginiana* | USA | OL435554 | OL435552 | OL439179 | Achatz et al. (2022a) |
| **Family Echinochasmidae** | | | | | | | |
| *Echinochasmus beleocephalus* | A | *Ardea alba* | Ukraine | KT956929 | – | – | Tkach et al. (2016) |
| *Echinochasmus coaxatus* | A | *Podiceps nigricollis* | Ukraine | KT956928 | – | – | Tkach et al. (2016) |
| *Echinochasmus donaldsoni* | A | *Podilymbus podiceps* | Ukraine | KT956930 | – | – | Tkach et al. (2016) |
| *Echinochasmus japonicus* | A | *Homo sapiens* | Vietnam | – | OR509030 | – | Le et al. (2024) |
| *Echinochasmus milvi* | A | ‘Duckling’ | Russia | – | MT447047 | – | Tatonova et al. (2020) |
| *Stephanoprora amurensis* | C | *Bulinus tropicus* | Zimbabwe | – | PP564877 | PP556555 | Mudavanhu et al. (2024) |
| *Stephanoprora amurensis* | A | *Phalacrocorax carbo* | Tanzania | – | MZ412884–MZ412885 | MZ394505–MZ394506 | Chibwana and Katandukila (2021) |
| *Stephanoprora amurensis* | A | *Gallus gallus* dom. | Russia | – | MT447043 | – | Tatonova et al. (2020) |
| **Family Echinostomatidae** | | | | | | | |
| *Drepanocephalus auratus* | C | *Planorbella trivolis* | USA | – | – | KP053262* | Pinto et al. (2016) |
| *Echinostoma bolschewense* | M | *Dreissena polymorpha* | Russia | – | – | MZ572871 | Bespalaya et al. (2022) |
| *Echinostoma miyagawai* | A | ‘duck’ | China | – | – | MH748721 | Li and Wang (2018) |
| Echinostomata sp.  Type II RS-2020 | C | *Bulinus globosus* | Zimbabwe | – | – | MT994273 | Schols et al. (2020) |
| Echinostomata sp.  Type Iia RS-2020 | C | *Bulinus globosus* | Zimbabwe | – | – | MT994274 | Schols et al. (2020) |
| Echinostomata sp.  III RS-2020 | C | *Biomphalaria pfeifferi* | Zimbabwe | – | – | MT994275 | Schols et al. (2020) |
| *Isthmiophora hortensis* | A | *Procyon lotor* | Japan | AB189982 | AB189982 | – | Sato et al. (2006) |
| *Isthmiophora melis* | A | *Apodemus agrarius* | Poland | KT359583 | – | – | Hildebrand et al. (2015) |
| *Isthmiophora melis* | C | *Lymnaea stagnalis* | Denmark |  | MW001033 | – | Duan et al. (2021) |
| *Isthmiophora* sp.  MN-2021 | R | *Radix auricularia* | Japan | LC599514 | – | – | Nakao and Sasaki (2021) |
| *Isthmiophora* sp.  VVT-2015 | A | *Neovison vison* | USA | KT956920 | – | – | Tkach et al. (2016) |
| *Neopetasiger islandicus* | C | *Gyraulus* cf. *parvus* | Iceland | MZ409816 | – | – | Pantoja et al. (2021) |
| *Neopetasiger islandicus* | C | *Planorbula armigera* | Canada | KT831344 | – | – | Gordy et al. (2016) |
| *Patagifer bilobus* | A | *Eudocimus albus* | USA | – | – | PV944192 | Díaz González et al. (2025) |
| *Patagifer paganoi* | A | *Theristicus caerulescens* | Argentina | – | – | PV944185 | Díaz González et al. (2025) |
| *Patagifer vioscai* | A | *Ardea cinerea* | Tanzania | – | – | MZ394510 | Chibwana and Katandukila (2021) |
| *Pegosomum asperum* | A | *Ardea alba* | Germany | – | KY945919 | – | Hirzmann (2017) |
| *Pegosomum saginatum* | A | *Ardea alba* | Germany | – | KY945918 | – | Hirzmann (2017) |
| *Petasiger exaeretus* | A | *Phalacrocorax carbo* | Hungary | KY284007–KY284009 | – | – | Cech et al. (2017) |
| *Petasiger exaeretus* | A | *Phalacrocorax carbo* | Hungary | – | PP188698 | – | Gyöngy et al. (2024) |
| *Petasiger exaeretus* | A | *Phalacrocorax carbo* | Hungary | – | KY283998 | – | Cech et al. (2017) |
| *Petasiger islandicus* | R | *Gyraulus* cf. *laevis* | Iceland | JQ425592 | – | – | Georgieva et al. (2012) |
| *Petasiger phalacrocoracis* | S | *Radix plicatula* | Japan | LC818882 | – | – | Seo et al. (2024) |
| *Petasiger phalacrocoracis* | M | *Rutilus rutilus* | Hungary | KY284004 | – | – | Cech et al. (2017) |
| *Petasiger phalacrocoracis* | M | *Scardinius erythrophthalmus* | Hungary | KY284005 | – | – | Cech et al. (2017) |
| *Petasiger phalacrocoracis* | A | *Phalacrocorax carbo* | Ukraine | KT956926 | – | – | Tkach et al. (2016) |
| *Petasiger phalacrocoracis* | A | *Phalacrocorax carbo* | Hungary | KY284006 | – | – | Cech et al. (2017) |
| *Petasiger phalacrocoracis* | A | *Phalacrocorax carbo* | Israel | – | AY245709 | – | Dzikowski et al. (2004) |
| *Petasiger phalacrocoracis* | A | *Phalacrocorax carbo* | Hungary | – | PP188697 | – | Gyöngy et al. (2024) |
| *Petasiger phalacrocoracis* | C | *Ampullaceana balthica* | Denmark | – | MW001053 | – | Duan et al. (2021) |
| *Petasiger phalacrocoracis* | M | *Rutilus rutilus* | Hungary | – | KJ720683 | – | Molnar et al. (2015) |
| *Petasiger phalacrocoracis* | A | *Phalacrocorax carbo* | Tanzania | – | MZ412883 | MZ394507–MZ394509 | Chibwana and Katandukila (2021) |
| *Petasiger radiatus* | C | *Burnupia transvaalensis* | South Africa | PP738959–PP738960 | PP738869 | – | Outa and Avenant-Oldewage (2024) |
| *Petasiger radiatus* | A | *Phalacrocorax carbo* | Ukraine | KT956927 | – | – | Tkach et al. (2016) |
| *Petasiger radiatus* | A | *Phalacrocorax carbo* | Hungary | KY284010 | – | – | Cech et al. (2017) |
| *Petasiger radiatus* | A | *Phalacrocorax carbo* | Israel | – | AY245708 | – | Dzikowski et al. (2004) |
| *Petasiger radiatus* | A | *Phalacrocorax carbo* | Hungary | – | KM973000 | – | Molnar et al. (2015) |
| *Petasiger radiatus* | A | *Phalacrocorax carbo* | Hungary | – | PP188703 | – | Gyöngy et al. (2024) |
| *Petasiger* sp. 1  ML-2019 | C | *Radix natalensis* | Kenya | MK482443 | – | MK534361* | Laidemitt et al. (2019) |
| *Petasiger* sp. 1  AK-2014 | C | *Planorbis planorbis* | Czech Republic | KM191800 | – | – | Selbach et al. (2014) |
| *Petasiger* sp. 1  AK-2014 | C | *Gyraulus albus* | Germany | KM191799 | – | – | Selbach et al. (2014) |
| *Petasiger* sp. 2  ML-2019 | C | *Bulinus globosus* | Kenya | MK482449–MK482450 | – | MK534366* | Laidemitt et al. (2019) |
| *Petasiger* sp. 2 ZA | R | *Pseusosuccinea columella* | South Africa | – | PP228840–PP228841 | – | Outa et al. (2024) |
| *Petasiger* sp. 2 ZA | C | *Radix natalensis* | South Africa | PP228374 | – | – | Outa et al. (2024) |
| *Petasiger* sp. 3  ML-2019 | C | *Bulinus* sp. | Kenya | MK482458 | – | MK534375* | Laidemitt et al. (2019) |
| *Petasiger* sp. 3  ML-2019 | C | *Radix natalensis* | Kenya | MK482446 | – | MK534364* | Laidemitt et al. (2019) |
| *Petasiger* sp. 3 ML | C | *Bulinus tropicus* | Uganda | – | – | OQ574001* | Hammoud et al. (2025) |
| *Petasiger* sp. 3 ZA | R | *Burnupia transvaalensis* | South Africa | PP738961–PP738962 | PP738870 | – | Outa and Avenant-Oldewage (2024) |
| *Petasiger* sp. 3 ZA | C | *Burnupia transvaalensis* | South Africa | PP738963 | – | PP738976–PP738977 | Outa and Avenant-Oldewage (2024) |
| *Petasiger* sp. 4  ML-2019 | C | *Biomphalaria pfeifferi* | Kenya | MK482430 | – | MK534350* | Laidemitt et al. (2019) |
| *Petasiger* sp. 4  ML-2019 | C | *Biomphalaria sudanica* | Kenya | MK482494 | – | MK534379* | Laidemitt et al. (2019) |
| *Petasiger* sp. 4  ML-2019 | C | *Biomphalaria sudanica* | Kenya | MK482467 | – | – | Laidemitt et al. (2019) |
| *Petasiger* sp. 4  ML-2019 | C | *Biomphalaria sudanica* | Kenya | MK482463 | – | – | Laidemitt et al. (2019) |
| *Petasiger* sp. 4  MAG-2019 | C | *Planorbella trivolvis* | Canada | – | – | MH369311* | Gordy and Hanington (2019) |
| *Petasiger* sp. 5  ML-2019 | C | *Bulinus globosus* | Kenya | MK482455 | – | MK534372* | Laidemitt et al. (2019) |
| *Petasiger* sp. 5  ML-2019 | C | *Bulinus truncatus trigonus* | Kenya | MK482480 | – | MK534396* | Laidemitt et al. (2019) |
| *Petasiger* sp. 5  ML-2019 | C | *Bulinus ugandae* | Kenya | – | – | MK534411* | Laidemitt et al. (2019) |
| *Petasiger* sp. 5  ML-2019 | C | *Bulinus* sp. | Kenya | MK482414 | – | MK534340* | Laidemitt et al. (2019) |
| *Petasiger* sp. 5  ML-2019 | C | *Bulinus* sp. | Kenya | MK482436 | – | – | Laidemitt et al. (2019) |
| *Petasiger* sp. 5  ML-2019 | C | *Bulinus* sp. | Kenya | MK482425 | – | – | Laidemitt et al. (2019) |
| *Petasiger* sp. 5 ML | C | *Bulinus tropicus* | Uganda | – | – | OQ543542 | Hammoud et al. (2025) |
| *Petasiger* sp. 5 ML | C | *Bulinus tropicus* | Uganda | – | – | OQ543549 | Hammoud et al. (2025) |
| *Petasiger* sp. 5 ML | C | *Bulinus tropicus* | Uganda | – | – | OQ543550 | Hammoud et al. (2025) |
| *Petasiger* sp. 5 ML | C | *Bulinus tropicus* | Uganda | – | – | OQ543551 | Hammoud et al. (2025) |
| *Petasiger* sp. 5 ML | C | *Bulinus tropicus* | Uganda | – | – | OQ574003* | Hammoud et al. (2025) |
| *Petasiger* sp. 5  ML-2019 | C | *Bulinus tropicus* | Uganda | – | – | ON970211* | Hammoud et al. (2022) |
| ***Petasiger* sp. 5 AH-2024** | **C** | ***Bulinus* sp.** | **South Africa** | **PX759001** | – | **PX775915** | **This study** |
| ***Petasiger* sp. 5 AH-2024** | **C** | ***Bulinus* sp.** | **South Africa** | – | **PX759006** | **PX775914/ PX763560*** | **This study** |
| ***Petasiger* sp. 5 AH-2024** | **C** | ***Bulinus* sp.** | **South Africa** | **PX759002** | – | **PX775916** | **This study** |
| ***Petasiger* sp. 5  AH-2024** | **C** | ***Bulinus* sp.** | **South Africa** | – | – | **PX775917** | **This study** |
| ***Petasiger* sp. 5  AH-2024** | **C** | ***Bulinus* sp.** | **South Africa** | – | – | **PX775918** | **This study** |
| ***Petasiger* sp. 5  AH-2024** | **C** | ***Bulinus* sp.** | **South Africa** | – | – | **PX775920** | **This study** |
| ***Petasiger* sp. 5 AH-2025** | **C** | ***Bulinus* sp.** | **South Africa** | **PX758998** | – | **PX775911** | **This study** |
| ***Petasiger* sp. 5 AH-2025** | **C** | ***Bulinus* sp.** | **South Africa** | **PX758999** | – | **PX775912** | **This study** |
| ***Petasiger* sp. 5 AH-2022** | **M** | ***Enteromius oraniensis*** | **South Africa** | **PX759004** | – | – | **This study** |
| *Petasiger* sp. 6 ML-2019 | C | *Bulinus* sp. | Kenya | MK482447 | – | – | Laidemitt et al. (2019) |
| *Petasiger* sp.  85_1 SS-2022 | C | *Isidorella hainesii* | Australia | – | OM305105 | – | Barton et. al. (2022) |
| *Petasiger* sp.  GC-2015 | M | *Rutilus rutilus* | Hungary | KY284002–KY284003 | KM972994–KM972995 | – | Cech et al. (2017) |
| *Petasiger* sp.  SS-2022 | C | *Bullastra lessoni* | Australia | – | ON866948 | – | Shamsi et al. (2024) |
| **Family Psilostomidae** | | | | | | | |
| Psilostomidae sp. RS-2020 | C | *Bulinus truncatus* | Zimbabwe | – | – | MT013353 | Schols et al. (2020) |
| **Family Strigeidae** | | | | | | | |
| *Apatemon gracilis* | C | *Ampullaceana balthica* | Norway | KY513175 | – | – | Soldánová et al. (2017) |
| *Apatemon* sp. ‘jamiesoni’ | C | *Potamopyrgus antipodarum* | New Zealand | – | – | KT334181 | Blasco-Costa et al. (2016) |
| *Apharyngostrigea cornu* | – | *Pseudodiplodiscus cornu* | – | AF184264 | – | – | Tkach et al. (2001) |
| *Apharyngostrigea cornu* | A | *Nycticorax nycticorax* | Mexico | – | JX977839 | – | Hernández-Mena et al. (2014) |
| *Australapatemon burti* | A | *Anas cyanoptera* | Mexico | – | JX977786 | – | Hernández-Mena et al. (2014) |
| *Australapatemon burti* | C | *Stagnicola elodes* | Canada | – | – | KY207576 | Gordy et. al. (2017) |
| *Australapatemon niewiadomski* | A | *Anas platyrhynchos* | New Zealand | – | – | KT334180 | Blasco-Costa et al. (2016) |
| *Parastrigea diovadena* | A | *Eudocimus albus* | Mexico | MF398348 | – | – | Hernández-Mena et al. (2017) |
| *Parastrigea diovadena* | A | *Eudocimus albus* | Mexico | – | JX977807 | – | Hernández-Mena et al. (2014) |
| **Gastropoda: Planorbidae** | | | | | | | |
| *Bulinus africanus* | – | – | South Africa | – | – | AM286296 | Kane et al. (2008) |
| *Bulinus barthi* | – | – | Tanzania | – | – | AM921814 | Kane et al. (2008) |
| *Bulinus forskalii* | – | – | Senegal | – | – | AM286307 | Kane et al. (2008) |
| *Bulinus globosus* | – | – | Kenya | – | – | AM921847 | Kane et al. (2008) |
| *Bulinus nasutus* | – | – | Uganda | – | – | AM921815 | Kane et al. (2008) |
| *Bulinus natalensis* | – | – | South Africa | – | – | AM921836 | Kane et al. (2008) |
| *Bulinus tropicus* | – | – | Uganda | – | – | MN551516 | Tumwebaze et al. (2019) |
| *Bulinus tropicus* |  |  | Kenya |  |  | OP242174 | Babbitt et al. (2023) |
| *Bulinus tropicus* | – | – | Kenya | – | – | ON112314 | Tumwebaze et al. (2022) |
| *Bulinus truncatus* | – | – | Tanzania | – | – | MT707362 | Chibwana et al. (2020) |
| *Bulinus truncatus* | – | – | Uganda | – | – | ON112302 | Tumwebaze et al. (2022) |
| ***Bulinus truncatus*** | **–** | **–** | **South Africa** | **–** | **–** | **PX776008** | **This study** |
| ***Bulinus* sp. AH-2025** | **–** | **–** | **South Africa** | **–** | **–** | **PX776006** | **This study** |
| *Bulinus* sp. 1 FDC-2020 | – | – | Tanzania | – | – | MT707392 | Chibwana et al. (2020) |
| *Bulinus* sp. 2 | – | – | Tanzania | – | – | AM286298 | Kane et al. (2008) |
| *Bulinus* sp. 4  IT-2022 | – | – | Tanzania | – | – | ON112304 | Tumwebaze et al. (2022) |
| *Bulinus* sp. 6 | – | – | Uganda | – | – | AM921819 | Kane et al. (2008) |
| *Bulinus* sp. 7  LSH IT-2022 | – | – | Lesotho | – | – | ON112309, ON112310 | Tumwebaze et al. (2022) |
| ***Bulinus* sp. 7 AH-2025** | – | – | South Africa | – | – | **PX776007** | **This study** |
| *Bulinus* sp. 8  MTE IT-2022 | – | – | Uganda | – | – | ON112311 | Tumwebaze et al. (2022) |
| *Bulinus wrighti* | – | – | Oman | – | – | AM286318 | Kane et al. (2008) |
| *Indoplanorbis exustus* | – | – | Gabon | – | – | MH037073 | Mouahid et al. (2018) |
| *Indoplanorbis exustus* | – | – | Malaysia | – | – | GU451746 | Liu et al. (2010) |

**

**

**Fig S1** Maximum Likelihood (ML) phylogram of selected *cox*1 mtDNA sequences of *Bulinus* spp. (Gastropoda: Planorbidae). Outgroup: *Indoplanorbis exustus* (Planorbidae). Nodal support is given as bootstrap values (>70) for ML analyses and posterior probabilities (>0.90) for Bayesian Inference (BI) analyses. Sequences generated in the current study are presented in bold.

**

**

**Fig S2** Maximum Likelihood (ML) phylogram of selected *nad*1 mtDNA sequences of family *Petasiger* spp. Outgroup: *Drepanocephalus auritus* (Echinostomatidae). Nodal support is given as bootstrap values (>70) for ML analyses and posterior probabilities (>0.90) for Bayesian Inference (BI) analyses. Host and life cycle stages (C, cercaria) are indicated for *Petasiger* sp. 5 (shaded). Sequences generated in this study are presented in bold.

**

**

**Fig S3** Maximum Likelihood (ML) phylogram of selected 28S sequences of family Diplostomidae. Outgroups: *Apatemon gracilis*, *Apharyngostrigea cornu*, *Parastrigea diovadena* (Strigeidae). Nodal support is given as bootstrap values (>70) for ML analyses and posterior probabilities (>0.90) for Bayesian Inference (BI) analyses. Host and life cycle stages (C, cercaria; M, metacercaria) are indicated for *Tylodelphys* sp. 2 (shaded). Sequences generated in this study are presented in bold.

**References**

Achatz, T.J., Chermak, T.P., Martens, J.R., Woodyard, E.T., Rosser, T.G., Pulis, E.E., Weinstein, S.B., Mcallister, C.T., Kinsella, J.M., Tkach, V.V. (2022a). Molecular phylogeny supports invalidation of *Didelphodiplostomum* and *Pharyngostomoides* (Digenea: Diplostomidae) and reveals a *Tylodelphys* from mammals. Zool J Linn Soc, 196 (1), 124–136. <https://doi.org/10.1093/zoolinnean/zlab114>

Achatz, T.J., Martens, J.R., Kostadinova, A., Pulis, E.E., Orlofske, S.A., Bell, J.A., Fecchio, A., Oyarzún-Ruiz, P., Syrota, Y.Y., Tkach, V.V. (2022b). Molecular phylogeny of *Diplostomum*, *Tylodelphys*, *Austrodiplostomum* and *Paralaria* (Digenea: Diplostomidae) necessitates systematic changes and reveals a history of evolutionary host switching events. Int J Parasitol, 52 (1), 47–63. <https://doi.org/10.1016/j.ijpara.2021.06.002>

Babbitt, C.R., Laidemitt, M.R., Mutuku, M.W., Oraro, P.O., Brant, S.V., Mkoji, G.M., Loker, E.S. (2023). *Bulinus* snails in the Lake Victoria Basin in Kenya: Systematics and their role as hosts for schistosomes. PLOS NTDs, 17 (2), e0010752. <https://doi.org/10.1371/journal.pntd.0010752>

Barton, D.P., Zhu, X., Nuhoglu, A., Pearce, L., McLellan, M., Shamsi, S. (2022). Parasites of selected freshwater snails in the Eastern Murray Darling Basin, Australia. Int J Environ Res Public Health, 19 (12), 7236. <https://doi.org/10.3390/ijerph19127236>

Behrmann-Godel, J. (2013). Parasite identification, succession and infection pathways in perch fry (*Perca fluviatilis*): new insights through a combined morphological and genetic approach. Parasitology, 140 (4), 509–520. <https://doi.org/10.1017/S0031182012001989>

Bespalaya, Y., Kondakov, A., Travina, O., Khrebtova, I., Kropotin, A., Aksenova, O., Gofarov, M., Lyubas, A., Tomilova, A. (2022). First record of metacercariae trematodes *Opisthioglyphe ranae* (Digenea: Telorchiidae) and *Echinostoma bolschewense* (Digenea: Echinostomatidae) in *Dreissena polymorpha* (Bivalvia: Dreissenidae) from the Don and Volga river basins, Russia. Ecol Montenegrina. 54, 57–76. <https://doi.org/10.37828/em.2022.54.8>

Blasco-Costa, I., Poulin, R., Presswell, B. (2016). Species of *Apatemon* Szidat, 1928 and *Australapatemon* Sudarikov, 1959 (Trematoda: Strigeidae) from New Zealand: linking and characterising life cycle stages with morphology and molecules. Parasitol Res, 115 (1), 271–289. <https://doi.org/10.1007/s00436-015-4744-0>

Brabec, J., Kostadinova, A., Scholz, T., Littlewood, D.T. (2015). Complete mitochondrial genomes and nuclear ribosomal RNA operons of two species of *Diplostomum* (Platyhelminthes: Trematoda): a molecular resource for taxonomy and molecular epidemiology of important fish pathogens. Parasit Vectors, 8, 336. <https://doi.org/10.1186/s13071-015-0949-4>

Cech, G., Molnar, K., Székely, C. (2017). Molecular biological studies of adult and metacercarial stages of *Petasiger exaeretus* Dietz, 1909 (Digenea: Echinostomatidae). Acta Vet Hung, 65 (2), 198–207. <https://doi.org/10.1556/004.2017.020>

Chaudhary, A., Gupta, S., Tripathi, R., Singh, H.S. (2017a). Morphological and molecular analyses of *Tylodelphys* spp. metacercaria (Trematoda: Diplostomidae) from the vitreous humour of two freshwater fish species, *Channa gachua* (Ham.) and *Puntius sophore* (Ham.). Vet Parasitol, 244, 64–70. <https://doi.org/10.1016/j.vetpar.2017.07.016>

Chaudhary, A., Tripathi, R., Gupta, S., Shanker Singh, H.S. (2017b). First report on molecular evidence of *Tylodelphys cerebralis* (Diplostomulum cerebralis) Chakrabarti, 1968 (Digenea: Diplostomidae) from snakehead fish *Channa punctata*. Acta Parasitol, 62 (2), 386–392. <https://doi.org/10.1515/ap-2017-0046>

Chibwana, F.D., Blasco-Costa, I., Georgieva, S., Hosea, K.M., Nkwengulila, G., Scholz, T., Kostadinova, A. (2013). A first insight into the barcodes for African diplostomids (Digenea: Diplostomidae): brain parasites in *Clarias gariepinus* (Siluriformes: Clariidae). Infect Genet Evol, 17, 62–70. <https://doi.org/10.1016/j.meegid.2013.03.037>

Chibwana, F.D., Hosea, K.M., Nkwengulila, G. (unpublished). Identity and phylogeny of *Diplostomum* metacercariae infecting the cat *fish Clarias gariepinus* in Tanzania based on sequence divergence of ITS region.

Chibwana, F.D., Katandukila, J. (2021). Occurrence of Echinostomatoids (Platyhelminthes: Digenea) in Great Cormorant (*Phalacrocorax carbo*) and Grey Heron (*Ardea cinerea*): First Insights into the DNA Barcodes from Lake Victoria, Tanzania. Afr Zool, 56, 181 –191.

Chibwana, F.D., Nkwengulila, G., Locke, S.A., McLaughlin, J.D., Marcogliese, D.J. (2015). Completion of the life cycle of *Tylodelphys mashonense* (Sudarikov, 1971) (Digenea: Diplostomidae) with DNA barcodes and rDNA sequences. Parasitol Res, 114 (10), 3675–3682. <https://doi.org/10.1007/s00436-015-4595-8>

Chibwana, F.D., Tumwebaze, I., Mahulu, A., Sands, A., Albrecht, C. (2020). Assessing the diversity and distribution of potential intermediate hosts snails for urogenital schistosomiasis: *Bulinus* spp. (Gastropoda: Planorbidae) of Lake Victoria. Parasit Vectors, 13. <https://doi.org/10.1186/s13071-020-04281-1>

Díaz González, M.G., López-Hernández, D., Tkach, V.V., Drago, F., Chibwana, F.D., Laidemitt, M.R., Blanar, C.A., Núñez, V., Díaz, M.D., Gomez-Puerta, L.A., Locke, S.A. (2025). Molecular and morphological support for the synonymy of *Nephrostomum* and *Patagifer*: discovery of new species and broad geographic connections. Int J Parasitol, S0020-7519(25)00205-X. <https://doi.org/10.1016/j.ijpara.2025.10.008>

Duan, Y., Al-Jubury, A., Kania, P.W., Buchmann, K. (2021). Trematode diversity reflecting the community structure of Danish freshwater systems: molecular clues. Parasit Vectors, 14 (1), 43. <https://doi.org/10.1186/s13071-020-04536-x>

Dzikowski, R., Levy, M.G., Poore, M.F., Flowers, J.R., Paperna, I. (2004). Use of rDNA polymorphism for identification of Heterophyidae infecting freshwater fishes. Dis Aquat Organ, 59 (1), 35–41. <https://doi.org/10.3354/dao059035>

García-Varela, M., Sereno-Uribe, A.L., Pinacho-Pinacho, C.D., Hernández-Cruz, E., Pérez-Ponce de León, G. (2016). An integrative taxonomic study reveals a new species of *Tylodelphys* Diesing, 1950 (Digenea: Diplostomidae) in central and northern Mexico. J Helminthol, 90 (6), 668–679. <https://doi.org/10.1017/S0022149X15000917>

Georgieva, S., Kostadinova, A. Skirnisson, K. (2012). The life-cycle of *Petasiger islandicus* Kostadinova & Skirnisson, 2007 (Digenea: Echinostomatidae) elucidated with the aid of molecular data. Syst Parasitol, 82, 177–183. <https://doi.org/10.1007/s11230-012-9354-y>

Gordy, M.A., Hanington, P.C. (2019). A fine-scale phylogenetic assessment of digenean trematodes in central Alberta reveals we have yet to uncover their total diversity. Ecol Evol, 9 (6), 3153–3238. https://doi.org/10.1002/ece3.4939

Gordy, M.A., Kish, L., Tarrabain, M., Hanington, P.C. (2016). A comprehensive survey of larval digenean trematodes and their snail hosts in central Alberta, Canada. Parasitol Res, 115 (10), 3867–3880. <https://doi.org/10.1007/s00436-016-5152-9>

Gordy, M.A., Locke, S.A., Rawlings, T.A., Lapierre, A.R., Hanington, P.C. (2017). Molecular and morphological evidence for nine species in North American *Australapatemon* (Sudarikov, 1959): a phylogeny expansion with description of the zygocercous *Australapatemon* *mclaughlini* n. sp. Parasitol Res, 116 (8), 2181–2198. <https://doi.org/10.1007/s00436-017-5523-x>

Gyöngy, M., Juhász, L., Sellyei, B., Székely, C., Cech, G. (2024). Digenean trematodes (Trematoda: Digenea) parasitizing the digestive system of the great cormorant (*Phalacrocorax carbo*) in Hungary. Helminthologia, 61 (4), 308–316. https://doi.org/10.2478/helm-2024-0033

Haarder, S., Jørgensen, K., Kania, P.W., Skovgaard, A., Buchmann, K. (2013). Occurrence of *Diplostomum pseudospathaceum* Niewiadomska, 1984 and *D. mergi* Dubois, 1932 (Digenea: Diplostomidae) in Danish freshwater snails: ecological and molecular data. Folia Parasitol, 60 (2), 177-180. <https://doi.org/10.14411/fp.2013.020>

Hammoud, C., Kayenbergh, A., Tumusiime, J., Verschuren, D., Albrecht, C., Huyse, T., Van Bocxlaer, B. (2022). Trematode infection affects shell shape and size in *Bulinus tropicus*. Int J Parasitol Parasit Wildl, 18, 300–311. <https://doi.org/10.1016/j.ijppaw.2022.07.003>

Hammoud, C., Van Bocxlaer, B., Verschuren, D., Tumusiime, J., Albrecht, C., De Crop, W., Umba Tolo, C., Huyse, T. (2025). Agricultural land use and ensuing eutrophication both shape parasitic trematode communities in rural African lakes. Proc R Soc B, 292, 20250070. <https://doi.org/10.1098/rspb.2025.0070>

Hernández-Mena, D.I., García-Prieto, L., García-Varela, M. (2014). Morphological and molecular differentiation of *Parastrigea* (Trematoda: Strigeidae) from Mexico, with the description of a new species. Parasitol Int, 63 (2), 315–323. <https://doi.org/10.1016/j.parint.2013.11.012>

Hernández-Mena, D. I., García-Varela, M., Pérez-Ponce de León, G. (2017). Filling the gaps in the classification of the Digenea Carus, 1863: systematic position of the Proterodiplostomidae Dubois, 1936 within the superfamily Diplostomoidea Poirier, 1886, inferred from nuclear and mitochondrial DNA sequences. Syst Parasitol, 94 (8), 833–848. <https://doi.org/10.1007/s11230-017-9745-1>

Hildebrand, J., Adamczyk, M., Laskowski, Z., Zaleśny, G. (2015). Host-dependent morphology of *Isthmiophora melis* (Schrank, 1788) Luhe, 1909 (Digenea, Echinostomatinae) – morphological variation vs. molecular stability. Parasit Vectors, 8, 481. <https://doi.org/10.1186/s13071-015-1095-8>

Hirzmann, J. (2017). Direct submission.

Ieshko, E., Lebedeva, D., Anikieva, L., Gorbach, V., Ilmast, N. (2022). Helminth communities of *Coregonus lavaretus* (Salmonidae: Coregoninae) from Lake Kamennoye (Kostomuksha State Nature Reserve, Russia). Nat Conserv Res, 7, <https://doi.org/10.24189/ncr.2022.032>

Kanarek, G., Gabrysiak, J., Pyrka, E., Jeżewski, W., Stanicka, A., Cichy, A., Żbikowska, E., Zaleśny, G., Hildebrand, J. (2023). Hyperparasitism among larval stages of Digenea in snail hosts: sophisticated life strategy or pure randomness? The scenario of *Cotylurus* sp. Zool J Linn Soc. <https://doi.org/10.1093/zoolinnean%2Fzlad102>

Kane, R.A., Stothard, J.R., Emery, A.M., Rollinson, D. (2008). Molecular characterization of freshwater snails in the genus *Bulinus*: a role for barcodes?. Parasit Vectors, 1 (1), 15. <https://doi.org/10.1186/1756-3305-1-15>

Karami, A.M., Marnis, H., Korbut, R., Zuo, S., Jaafar, R., Duan, Y., Mathiessen, H., Al-Jubury, A., Kania, P.W., Buchmann, K. (2022). Absence of zoonotic parasites in salmonid aquaculture in Denmark: Causes and consequences. Aquaculture, 549, 737793. <https://doi.org/10.1016/j.aquaculture.2021.737793>

Kudlai, O., Rakauskas, V., Baker, N.J., Pantoja, C., Lisitsyna, O., Binkienė, R. (2024). Helminth parasites of invasive freshwater fish in Lithuania. Animals, 14 (22), 3293. <https://doi.org/10.3390/ani14223293>

Kundid, P., Pantoja, C., Soldanova, M. (2025). Timing matters: exploring emergence patterns of two species of trematode furcocercariae from their snail hosts. Folia Parasitol, 72, 2025.008. <https://doi.org/10.14411/fp.2025.008>

Laidemitt, M.R., Brant, S.V., Mutuku, M.W., Mkoji, G.M., Loker, E.S. (2019). The diverse echinostomes from East Africa: With a focus on species that use *Biomphalaria* and *Bulinus* as intermediate hosts. Acta Trop, 193, 38–49. <https://doi.org/10.1016/j.actatropica.2019.01.025>

Le, T.H., Pham, L.T.K., Van Quyen, D., Nguyen, K.T., Doan, H.T.T., Saijuntha, W., Blair, D. (2024). The ribosomal transcription units of five echinostomes and their taxonomic implications for the suborder Echinostomata (Trematoda: Platyhelminthes). Parasitol Res, 123 (1), 103. <https://doi.org/10.1007/s00436-023-08110-z>

Li, Y., Wang, C.R. (2018). Direct submission.

Liu, L., Mondal, M.M., Idris, M.A., Lokman, H.S., Rajapakse, P.J., Satrija, F., Diaz, J.L., Upatham, E.S., Attwood, S.W. (2010). The phylogeography of *Indoplanorbis exustus* (Gastropoda: Planorbidae) in Asia. Parasit Vectors, (3) 57. <https://doi.org/10.1186/1756-3305-3-57>

Locke, S.A., Al-Nasiri, F.S., Caffara, M., Drago, F., Kalbe, M., Lapierre, A.R., McLaughlin, J.D., Nie, P., Overstreet, R.M., Souza, G.T., Takemoto, R.M., Marcogliese, D.J. (2015). Diversity, specificity and speciation in larval Diplostomidae (Platyhelminthes: Digenea) in the eyes of freshwater fish, as revealed by DNA barcodes. Int J Parasitol, 45 (13), 841–855. <https://doi.org/10.1016/j.ijpara.2015.07.001>

Locke, S.A., Van Dam, A., Caffara, M., Pinto, H.A., López-Hernández, D., Blanar, C.A. (2018). Validity of the Diplostomoidea and Diplostomida (Digenea, Platyhelminthes) upheld in phylogenomic analysis. Int J Parasitol, 48(13), 1043–1059. <https://doi.org/10.1016/j.ijpara.2018.07.001>

López-Jiménez, A., Pérez-Ponce de León, G., García-Varela, M. (2018). Molecular data reveal high diversity of *Uvulifer* (Trematoda: Diplostomidae) in Middle America, with the description of a new species. J Helminthol, 92 (6), 725–739. <https://doi.org/10.1017/S0022149X17000888>

Maraganga, J.M., Rindoria, N.M., Morara, G.M., Kimani, V.N., Wyk, C.V., Dumbo, J.C., Smit, N.J., Luus-Powell, W.J. (2023). Tegumental topography and molecular characterisation of two trematodes (Platyhelminthes: Digenea) from *Clarias gariepinus* (Burchell, 1822) in Kenya. Int J Parasitol Parasites Wildl, 23, 100897. <https://doi.org/10.1016/j.ijppaw.2023.100897>

Martinek, I., Hernández-Orts, J.S. (2023). Helminth fauna of the black goby *Gobius niger* L. (Gobiiformes: Gobiidae) from the Finnish Archipelago, Baltic Sea: Molecular and morphological data. Curr Res Parasitol Vector Borne Dis, 5, 100169. <https://doi.org/10.1016/j.crpvbd.2023.100169>

Mehrdana, F., Marana, M.H., Skov, J., Bahlool, Q.Z., Sindberg, D., Mundeling, M., Overgaard, B.C., Kania, P.W., Buchmann, K. (2015). Eye fluke infection status in Baltic cod, *Gadus morhua*, after three decades and their use as ecological indicators. Acta Parasitol, 60 (3), 423–429. <https://doi.org/10.1515/ap-2015-0058>

Moema, E.B., King, P.H., Rakgole, J.N., Baker, C. (2013). Descriptions of diplostomid metacercariae (Digenea: Diplostomidae) from freshwater fishes in the Tshwane area. OJVR, 80 (1), 611. <https://doi.org/10.4102/ojvr.v80i1.611>

Molnar, K., Gibson, D.I., Cech, G., Papp, M., Deak-Paulus, P., Juhasz, L., Toth, N., Szekely, C. (2015). The occurrence of metacercariae of *Petasiger* (Digenea: Echinostomatidae) in an unusual site, within the lateral line scales of cyprinid fishes. Folia Parasitol, 62, 2015.017. <https://doi.org/10.14411/fp.2015.017>

Moszczynska, A., Locke, S.A., McLaughlin, J.D., Marcogliese, D.J., Crease, T.J. (2009). Development of primers for the mitochondrial cytochrome c oxidase I gene in digenetic trematodes (Platyhelminthes) illustrates the challenge of barcoding parasitic helminths. Mol Ecol Resour, 9 Suppl s1, 75–82. <https://doi.org/10.1111/j.1755-0998.2009.02634.x>

Mouahid, G., Clerissi, C., Allienne, J-F., Chaparro, C., Yafae, S., Mintsa Nguema, R., Ibikounlé, M., Moné, H. (2018). The phylogeny of the genus *Indoplanorbis* (Gastropoda, Planorbidae) from Africa and the French West Indies. Zool Scr, 47. <https://doi.org/10.1111/zsc.12297?urlappend=%3Futm_source%3Dresearchgate.net%26utm_medium%3Darticle>

Mudavanhu, A., Schols, R., Goossens, E., Nhiwatiwa, T., Manyangadze, T., Brendonck, L., Huyse, T. (2024). One Health monitoring reveals invasive freshwater snail species, new records, and undescribed parasite diversity in Zimbabwe. Parasit Vectors 17 (1), 234. https://doi.org/10.1186/s13071-024-06307-4

Nakao, M., Sasaki, M. (2021). Trematode diversity in freshwater snails from a stopover point for migratory waterfowls in Hokkaido, Japan: An assessment by molecular phylogenetic and population genetic analyses. Parasitol Int, 83, 102329. <https://doi.org/10.1016/j.parint.2021.102329>

Otachi, E.O., Locke, S.A., Jirsa, F., Fellner-Frank, C., Marcogliese, D.J. (2015). Morphometric and molecular analyses of *Tylodelphys* sp. metacercariae (Digenea: Diplostomidae) from the vitreous humour of four fish species from Lake Naivasha, Kenya. J Helminthol, 89 (4), 404–414. <https://doi.org/10.1017/S0022149X14000170>

Outa, J.O., Avenant-Oldewage, A. (2024). Echinostomatids from South African freshwater limpets: phylogenetic analyses and diagnostic morphological features for cercariae of *Petasiger*. J Helminthol, 98, e91. <https://doi.org/10.1017/S0022149X24000749>

Outa, J.O., Bhika, P., Avenant-Oldewage, A. (2024). Gastropod invasions in anthropogenically impacted impoundments in South Africa: Tracing their origins and exploring field evidence of parasite spillback and amplification. Int J Parasitol, 54 (6), 279–301. <https://doi.org/10.1016/j.ijpara.2024.02.004>

Pantoja, C., Faltýnková, A., O'Dwyer, K., Jouet, D., Skírnisson, K., Kudlai, O. (2021). Diversity of echinostomes (Digenea: Echinostomatidae) in their snail hosts at high latitudes. Parasite 28, 59. <https://doi.org/10.1051/parasite/2021054>

Pinto, H.A., Griffin, M.J., Quiniou, S.M., Ware, C., Melo, A.L. (2016). *Biomphalaria straminea* (Mollusca: Planorbidae) as an intermediate host of *Drepanocephalus* spp. (Trematoda: Echinostomatidae) in Brazil: a morphological and molecular study. Parasitol Res, 115 (1), 51–62. https://doi.org/10.1007/s00436-015-4469-0

Sato, H., Suzuki, K. (2006). Gastrointestinal helminths of feral raccoons (*Procyon lotor*) in Wakayama Prefecture, Japan. J Vet Med Sci, 68 (4), 311–318. <https://doi.org/10.1292/jvms.68.311>

Schols, R., Mudavanhu, A., Carolus, H., Hammoud, C., Muzarabani, K. C., Barson, M., Huyse, T. (2020). Exposing the Barcoding Void: An Integrative Approach to Study Snail-Borne Parasites in a One Health Context. Front Vet Sci, 7, 605280. <https://doi.org/10.3389/fvets.2020.605280>

Schwelm, J., Georgieva, S., Grabner, D.S., Kostadinova, A., Sures, B. (2021). Molecular and morphological characterisation of *Diplostomum phoxini* (Faust, 1918) with a revised classification and an updated nomenclature of the species-level lineages of *Diplostomum* (Digenea: Diplostomidae) sequenced worldwide. Parasitology, 148, 1648–1664. <https://doi.org/10.1017/S0031182021001372>

Selbach, C., Soldánová, M., Georgieva, S., Kostadinova, A., Kalbe, M., Sures, B. (2014). Morphological and molecular data for larval stages of four species of *Petasiger* Dietz, 1909 (Digenea: Echinostomatidae) with an updated key to the known cercariae from the Palaearctic. Syst Parasitol, 89, 153-66. <https://doi.org/10.1007/s11230-014-9513-4>

Seo, H., Ansai, E., Sase, T., Saito, T., Takano, T., Kojima, Y., Waki, T. (2024). Introduction of a snake trematode of the genus *Ochetosoma* in eastern Japan. Parasitol Int, 103, 102947. <https://doi.org/10.1016/j.parint.2024.102947>

Shamsi, S., Banfield, A., Francis, N., Barton, D.P., McLellan, M. (2024). Characterisation of Nematoda and Digenea in selected Australian freshwater snails. J Invertebr Pathol, 204, 108116. <https://doi.org/10.1016/j.jip.2024.108116>

Soldánová, M., Georgieva, S., Roháčová, J., Knudsen, R., Kuhn, J.A., Henriksen, E.H., Siwertsson, A., Shaw, J.C., Kuris, A.M., Amundsen, P.A., Scholz, T., Lafferty, K.D., Kostadinova, A. (2017). Molecular analyses reveal high species diversity of trematodes in a sub-Arctic lake. Int J Parasitol, 47 (6), 327–345. <https://doi.org/10.1016/j.ijpara.2016.12.008>

Svinin, A.O., Chikhlyaev, I.V., Bashinskiy, I.W., Osipov, V.V., Neymark, L.A., Ivanov, A.Y., Stoyko, T.G., Chernigova, P.I., Ibrogimova, P.K., Litvinchuk, S.N., Ermakov, O.A. (2023). Diversity of trematodes from the amphibian anomaly P hotspot: Role of planorbid snails. PLOS ONE, 18 (3), e0281740. <https://doi.org/10.1371/journal.pone.0281740>

Tatonova, Y.V., Izrailskaia, A.V., Besprozvannykh, V.V. (2020). *Stephanoprora* *amurensis* sp. nov., *Echinochasmus milvi* Yamaguti, 1939 and *E. suifunensis* Besprozvannykh, 1991 from the Russian southern Far East and their phylogenetic relationships within the Echinochasmidae Odhner 1910. Parasitology, 147 (13), 1469–1479. <https://doi.org/10.1017/S0031182020001444>

Tkach, V.V., Kudlai, O., Kostadinova, A. (2016). Molecular phylogeny and systematics of the Echinostomatoidea Looss, 1899 (Platyhelminthes: Digenea). Int J Parasitol, 46 (3), 171–185. <https://doi.org/10.1016/j.ijpara.2015.11.001>

Tkach, V.V., Pawlowski, J., Mariaux, J., Swiderski, Z. (2001). Molecular phylogeny of the suborder Plagiorchiata and its position in the system of Digenea. In: Littlewood, D.T.J., Bray, R.A. (eds.). Interrelationships of the Platyhelminthes. Taylor & Francis, London.

Tumwebaze, I., Clewing, C., Chibwana, F.D., Kipyegon, J.K., Albrecht, C. (2022). Evolution and Biogeography of Freshwater Snails of the Genus *Bulinus* (Gastropoda) in Afromontane Extreme Environments. Front Environ Sci, 10, 902900. <https://doi.org/10.3389/fenvs.2022.902900>

Tumwebaze, I., Clewing, C., Dusabe, M.C., Tumusiime, J., Kagoro-Rugunda, G., Hammoud, C., Albrecht, C. (2019). Molecular identification of *Bulinus* spp. intermediate host snails of *Schistosoma* spp. in crater lakes of western Uganda with implications for the transmission of the *Schistosoma haematobium* group parasites. Parasit Vectors, 12 (1), 565. <https://doi.org/10.1186/s13071-019-3811-2>
